# Embryonic spinocerebellar ataxia type 37-associated AUUUC repeat RNA causes neurodevelopmental defects

**DOI:** 10.1242/dmm.052636

**Published:** 2026-04-30

**Authors:** Ana F. Castro, Ana S. Figueiredo, Joana R. Loureiro, Maria M. Azevedo, Paula Sampaio, Ana M. Valentim, José Bessa, Isabel Silveira

**Affiliations:** ^1^ Genetics of Cognitive Dysfunction Laboratory, IBMC-Institute for Molecular and Cell Biology and i3S-Institute for Research and Innovation in Health Sciences, University of Porto, Rua Alfredo Allen 208, 4200-135 Porto, Portugal; ^2^ ICBAS-School of Medicine and Biomedical Sciences Abel Salazar, University of Porto, Rua Jorge de Viterbo Ferreira 228, 4050-313 Porto, Portugal; ^3^ Advanced Light Microscopy Platform, IBMC-Institute for Molecular and Cell Biology and i3S-Institute for Research and Innovation in Health Sciences, University of Porto, Rua Alfredo Allen 208, 4200-135 Porto, Portugal; ^4^ Laboratory Animal Science, IBMC-Institute for Molecular and Cell Biology and i3S-Institute for Research and Innovation in Health Sciences, University of Porto, Rua Alfredo Allen 208, 4200-135 Porto, Portugal; ^5^ Vertebrate Development and Regeneration Laboratory, IBMC-Institute for Molecular and Cell Biology and i3S-Institute for Research and Innovation in Health Sciences, University of Porto, Rua Alfredo Allen 208, 4200-135 Porto, Portugal

**Keywords:** Pentanucleotide repeat disease, Intronic *DAB1* ATTTC repeat, Familial adult myoclonic epilepsy (FAME), NOVA2, Axonal outgrowth, Synaptic innervation defects

## Abstract

Onset of many neurodegenerative and neuromuscular diseases usually starts in adulthood; however, recent advances point towards neurodevelopmental changes as drivers of late neurodegeneration. How early neuropathological features occur under these conditions remains unclear, but this knowledge would be critical for timely therapeutic intervention. Here, we provide evidence that neurodevelopmental axonal defects initiate a motor phenotype in a zebrafish model of spinocerebellar ataxia type 37 (SCA37), a degenerative hereditary disease caused by an ATTTC repeat in the *DAB1* gene. We investigated neuronal defects triggered by the embryonic AUUUC repeat RNA from the *DAB1* gene and their effects later in life by transiently expressing this RNA in embryos and analyzing innervation and motor function. We found abnormalities in motor neuron axonal outgrowth and muscle innervation. We also discovered disrupted embryonic motor activity, and reduced locomotor distance and velocity in late adult zebrafish, demonstrating motor impairment. Moreover, we showed that protein expression of the splicing regulator NOVA2 rescues axonal defects, indicating dysfunction of NOVA2-regulated neurodevelopmental processes. Overall, our results establish embryonic expression of the AUUUC repeat RNA as a driver of axonal and synaptic abnormalities, interfering with neuronal circuits and culminating in adult motor dysfunction.

## Teaser

**Summary:** Spinocerebellar ataxia type 37 is a neurodegenerative disease, usually with late-adulthood onset. Using a zebrafish model, we provide evidence that neurodevelopmental synaptic defects may contribute to this disease.

## INTRODUCTION

Growing evidence from studies using mouse models of neurodegenerative diseases suggests that abnormal synaptic connectivity during the first days of development sets the stage for neurodegeneration (Capizzi et al., 2022; Edamakanti et al., 2018; Serra et al., 2006; Shabani and Hassan, 2023). This neurodevelopmental dysfunction has been particularly evident in spinocerebellar ataxia type 1 (SCA1), where early developmental expression of the mutant ataxin 1 (ATX1) protein leads to abnormal cerebellar connectivity (Edamakanti et al., 2018; Serra et al., 2006). Spinocerebellar ataxias (SCAs) are autosomal-dominant neurodegenerative diseases characterized by progressive motor incoordination and cerebellar Purkinje cell degeneration (Klockgether et al., 2019). They are frequently caused by an expanded short tandem repeat or microsatellite sequence within coding or non-coding gene regions, whose length partly explains the variability in age of disease onset (Loureiro et al., 2022). We have previously identified a primate-specific ATTTC DNA repeat that is inserted into a pre-existing polymorphic ATTTT repeat of an Alu retrotransposon located within the 5′ UTR intron 1 of the gene encoding DAB adaptor protein 1 (*DAB1*) as the cause of SCA37 (Seixas et al., 2017). While the non-pathogenic alleles may be even larger, ranging from 7 to 400 ATTTT repeat units, they never contain the pathogenic ATTTC repeat insertion (Seixas et al., 2017). *DAB1* is expressed during embryonic development and encodes the reelin adaptor protein DAB1 that is known to have an important role in neurodevelopment, including neuronal migration, brain layer organization and axonal pathfinding (Beffert et al., 2002; D'Arcangelo et al., 1995; Howell et al., 1997; Rice et al., 1998; Sheldon et al., 1997; Trommsdorff et al., 1999). Individuals diagnosed with SCA37 usually present a phenotype of cerebellar ataxia, including dysarthria, and gait and limb incoordination (Corral-Juan et al., 2018; Rosenbohm et al., 2022; Seixas et al., 2017); however, impairment of the lower motor neurons – similar as to other types of SCA (García-Murias et al., 2012; Ikeda et al., 2012; Klockgether et al., 2019) – has also been reported (Rosenbohm et al., 2022). Neuropathological analysis of post-mortem cerebellar tissue from SCA37 patients has shown loss of Purkinje cells, diminished thickness of the molecular layer and axonal defects (Corral-Juan et al., 2018). Additionally, mispositioning of Purkinje cell somas within the molecular and granular layers has been observed in brain tissue from elderly individuals diagnosed with SCA37 (Corral-Juan et al., 2018), implying impaired cerebellar neurodevelopment. This, together with our previous evidence of *in vivo* developmental toxicity of the SCA37-associated AUUUC repeat RNA (Seixas et al., 2017), raises the hypothesis that SCA37 may have an early developmental origin similar to what has been found in human fetuses carrying the Huntington's disease variant (Barnat et al., 2020). Notwithstanding the neuropathological characterization of elderly SCA37 individuals after many decades of disease progression, the embryonic impact of the AUUUC repeat RNA [(AUUUC)_n_ hereafter] remains poorly understood.

We have shown that the pathogenic *DAB1* sequence containing 58 ATTTC repeat units [(ATTTC)_58_] triggers nuclear RNA aggregation in human cells and causes lethality in zebrafish embryos upon (AUUUC)_58_ RNA microinjection, indicating a pathogenic RNA-mediated mechanism in SCA37 (Seixas et al., 2017). Nuclear (AUUUC)_n_ aggregates have been seen in cerebellar Purkinje cells and cortical neurons from brain tissue of affected individuals with familial adult myoclonic epilepsy (FAME) (Ishiura et al., 2018). These FAME disorders and SCA37 belong to a group of, currently, eight (ATTTC)_n_ insertion conditions (Corbett et al., 2019; Florian et al., 2019; Ishiura et al., 2018; Ishiura and Tsuji, 2020; Loureiro et al., 2022; Silveira and Bennett, 2023; Yeetong et al., 2024, 2019) that are likely to share an RNA-mediated mechanism. One of the pathogenic processes that seems to explain the cellular and neurological phenotypes associated with expanded RNA repeats is the abnormal binding and sequestration of RNA-binding proteins (RBPs), which trigger nuclear RNA aggregate formation and RBP loss-of-function (Loureiro et al., 2022; Zhang and Ashizawa, 2022). This mechanism contributes to several neurodegenerative conditions caused by non-coding repeat expansions, such as SCA37, which result from pathogenic RNA repeat expression (Ishiguro et al., 2017; Swinnen et al., 2018; Todd et al., 2014; White et al., 2010). We have shown sequestration of the RNA-binding protein Nova-2 (NOVA2) by pathogenic (AUUUC)_58_ RNA in human embryonic neural stem cells (Figueiredo et al., 2025 preprint), raising the hypothesis of a developmental NOVA2 loss-of-function in SCA37. NOVA2 is an RBP known to have major roles in human neurodevelopment, as its dysfunction has been associated with neurodevelopmental disorders (Mattioli et al., 2020; Scala et al., 2022). It regulates developmental axon guidance and synaptic circuit formation by controlling the alternative splicing of many neurodevelopmental genes (Ruggiu et al., 2009; Saito et al., 2016, 2019), including *DAB1* (Corral-Juan et al., 2018; Mattioli et al., 2020; Yano et al., 2010). The fetal expression of the AUUUC repeat within *DAB1* RNA (hereafter referred to as ‘AUUUC repeat RNA’) and its incontestable role in pathogenesis (Seixas et al., 2017) render the investigation of whether and how early neuropathological defects contribute to SCA37 fundamental to advancing knowledge on pathogenesis, and timely therapeutic intervention for pentanucleotide repeat and other similar neurodegenerative diseases.

In this study, we established that the embryonic expression of the SCA37-associated (AUUUC)_58_ RNA in zebrafish causes developmental axonal and synaptic defects and disrupts adult zebrafish locomotor function. In addition, we demonstrated that axonal defects are rescued by NOVA2, showing that NOVA2 dysfunction plays a role in SCA37. In summary, we show that the SCA37-associated AUUUC repeat caused developmental motor neuron pathology, potentially through NOVA2 loss-of-function, highlighting that early therapeutic intervention is key to mitigating this and similar pentanucleotide repeat disorders.

## RESULTS

### The pathogenic (AUUUC)_58_ RNA induces developmental defects in zebrafish

**Fig. 1.**
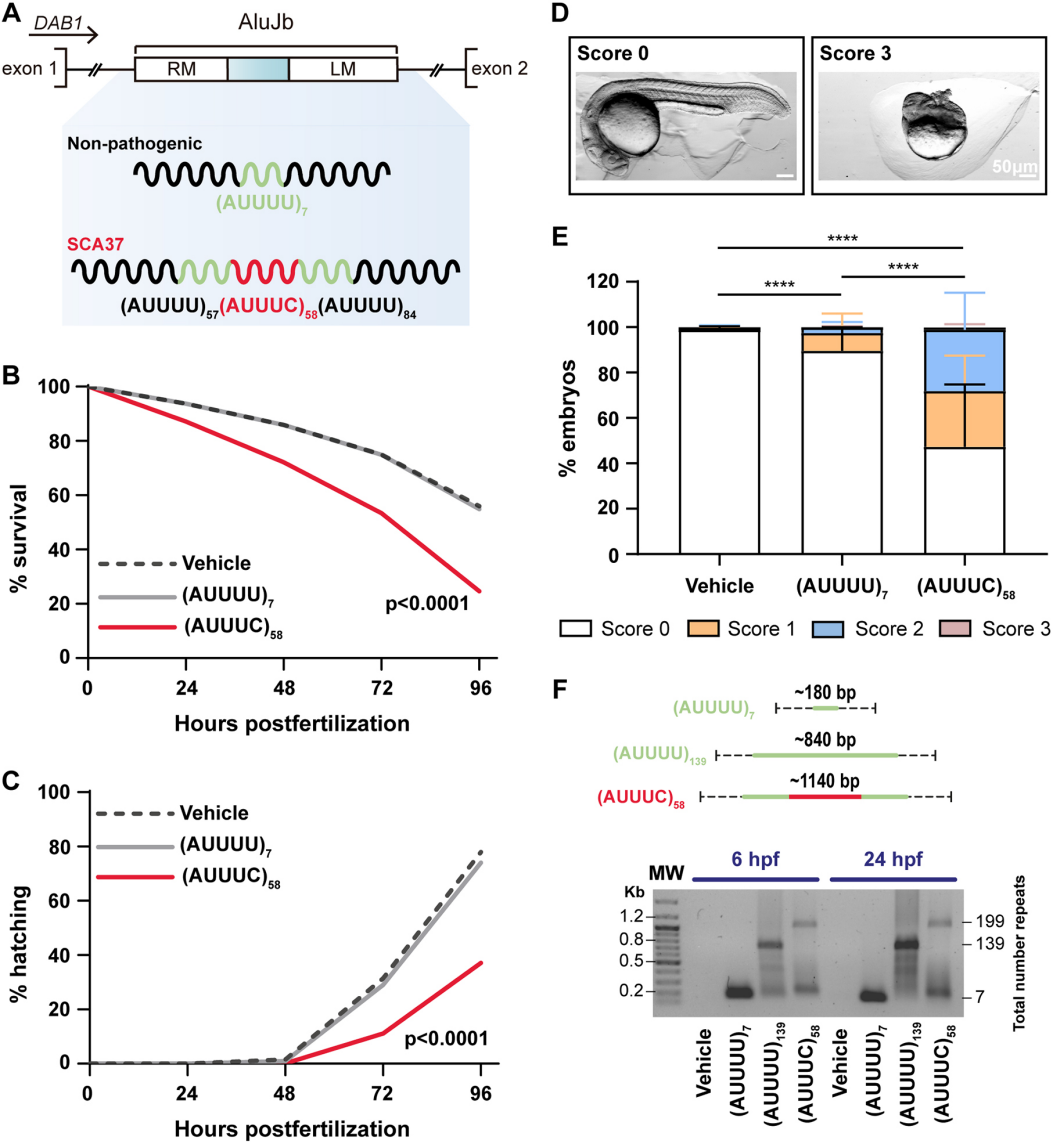
**SCA37-associated (AUUUC)_58_ RNA induces developmental defects in zebrafish.** Data obtained after embryo microinjection of vehicle, (AUUUU)_7_, or (AUUUC)_58_ RNAs; seven replicates; at least 100 embryos per condition. (A) Schematic of microinjected RNAs with non-pathogenic (AUUUU)_7_ or pathogenic SCA37-associated (AUUUC)_58_ insertion, along with AluJb flanking regions. (B) Plotted survival rates of zebrafish between 0 and 96 hpf microinjected as described in A (****P<0.0001, log-rank test). (C) Plotted hatching rate of zebrafish between 0 and 96 hpf microinjected as described in A (****P<0.0001, log-rank test). (D) Representative images showing a score-0 embryo similar to wild type (left) and a score-3 embryo with severe developmental arrest (right) (scale bars: 50 µm). (E) Plot showing the percentage of scored embryos at 24 hpf. Score 0 (no morphological defects; white), score 1 (tail defects; orange), score 2 (head and tail defects; blue), score 3 (developmental arrest; red) (****P<0.0001, Kruskal–Wallis with Dunn's post-hoc test). Data are presented as the mean+standard deviation. (F) Schematic of the expected repeat length of each RNA repeat (top). Agarose gel electrophoresis, showing RT-PCR-amplified RNA repeats at the expected sizes after microinjection with vehicle, (AUUUU)_7_, (AUUUU)_139_, or (AUUUC)_58_ RNAs at 6 and 24 hpf (bottom).

The primate-specific SCA37-associated (AUUUC)_n_ RNA, which is encoded in a *DAB1* intron, is more actively transcribed during embryonic development than in adulthood (Seixas et al., 2017). Therefore, to investigate whether the pathogenic (AUUUC)_n_ RNA affects early vertebrate development, we microinjected one- to two-cell-stage wild-type zebrafish embryos with the transcribed RNA repeats (Fig. 1A). These included the non-pathogenic (AUUUU)_7_, which is one of the most common alleles in the unaffected population and intergenerationally stable (Seixas et al., 2017), and the pathogenic (AUUUU)_57_(AUUUC)_58_(AUUUU)_84_ insertion, hereafter referred to as (AUUUC)_58_, transcribed RNAs. Both RNAs contain the flanking monomers of the AluJb element present in the *SCA37* locus, as illustrated in Fig. 1A. Then, we performed embryo phenotyping and found that the (AUUUC)_58_ RNA microinjection significantly increased the lethality and number of morphological developmental defects (77±21%) at 24 h post fertilization (hpf), compared with non-pathogenic (AUUUU)_7_ and vehicle (water-injected) counterparts (33±12% and 25±13%, respectively; *P*<0.0001) (Fig. S1A), reproducing our previous findings (Seixas et al., 2017). We also monitored the survival and hatching of microinjected embryos daily from 0 to 96 hpf. We detected a significantly decreased survival rate (25%) and number of hatched embryos (37%) in the (AUUUC)_58_-injected group compared with the non-pathogenic (AUUUU)_7_-injected (survival rate: 55%; hatched embryos: 74%; *P*<0.0001 for survival and hatching) or vehicle group (survival rate: 56%; hatched embryos: 78%; *P*<0.0001 for survival and hatching) (Fig. 1B,C). This demonstrates that the pathogenic (AUUUC)_58_ RNA decreases the survival of zebrafish embryos, delaying the hatching process. We further examined the morphology of the head and tail in surviving embryos at 24-26 hpf. We established a phenotypical score ranging from 0 to 3 (Fig. 1D and Fig. S1B), and quantified the number of embryos for each score, finding that the distribution of scores was significantly different between embryos microinjected with one of each, RNA or vehicle, (*P*<0.0001) (Fig. 1E and Fig. S1C). We found that the microinjection of the pathogenic (AUUUC)_58_ RNA significantly decreased the number of embryos with normal morphology (score 0: 47.22±27.48%), increased the presence of tail and/or head malformations (score 1: 24.63±15.61%; score 2: 26.99±16.28%) and caused developmental arrest of the zebrafish (score 3: 1.16±1.21%), compared with the non-pathogenic (AUUUU)_7_ RNA (score 0: 89.53±10.40%; score 1: 7.90±8.55%; score 2: 2.39±2.43%; score 3: 0.18±0.46%) or vehicle (score 0: 98.81±1.51%; score 1: 0.38±1.01%; score 2: 0.67±0.94%; score 3: 0.14±0.37%) microinjections (*P*<0.0001) (Fig. 1E and Fig. S1C). We further confirmed that the increase of developmental defects is caused by the (AUUUC)_58_ RNA and not by large AUUUU repeat RNAs. To test this, we performed the experiment described above with a large non-pathogenic (AUUUU)_139_ RNA (Fig. S2), derived from an allele present in unaffected members of SCA37 families reported previously (Seixas et al., 2017). To rule out injection efficiency variability, we co-microinjected embryos with each RNA repeat and *GFP* mRNA. At 24 hpf, nearly all microinjected embryos in each condition expressed GFP [vehicle: 98.67±1.53%; (AUUUU)_7_: 99.33±0.58%; (AUUUU)_139_: 99.67±0.58%; and (AUUUC)_58_: 98.83±1.26%] (Fig. S3A,B). Importantly, morphological defects were detected only in GFP-positive embryos (Fig. S3C). To assess the temporal profile of the human-specific transient RNA repeat after embryo microinjection, we extracted RNA from pooled embryos of each condition and amplified the repeat region by reverse transcription (RT)-PCR, observing for each condition the PCR amplification of a marked product corresponding to the size of the microinjected RNA repeat, at 6 and 24 hpf (Fig. 1F), which confirms their integrity at these time-points. The amplified PCR product remained detectable up to 120 hpf, albeit with considerable degradation at these later stages (Fig. S4). Because pronounced developmental abnormalities have not so far been found in SCA37 family members (Corral-Juan et al., 2018; Rosenbohm et al., 2022; Seixas et al., 2017) and to avoid indirect phenotypes arising from general developmental abnormalities, we selected score-0 embryos without visible developmental malformations. These embryos were staged at 24 hpf according to the established morphological criteria of body axis straightening from its early curvature around the yolk (Kimmel et al., 1995). We then used these embryos to characterize the impact of the pathogenic RNA on embryonic development, conducting the experiments described hereafter.

### Transient expression of the SCA37-associated (AUUUC)_58_ RNA in early zebrafish development does not impair zebrin II-positive Purkinje cell differentiation

**Fig. 2.**
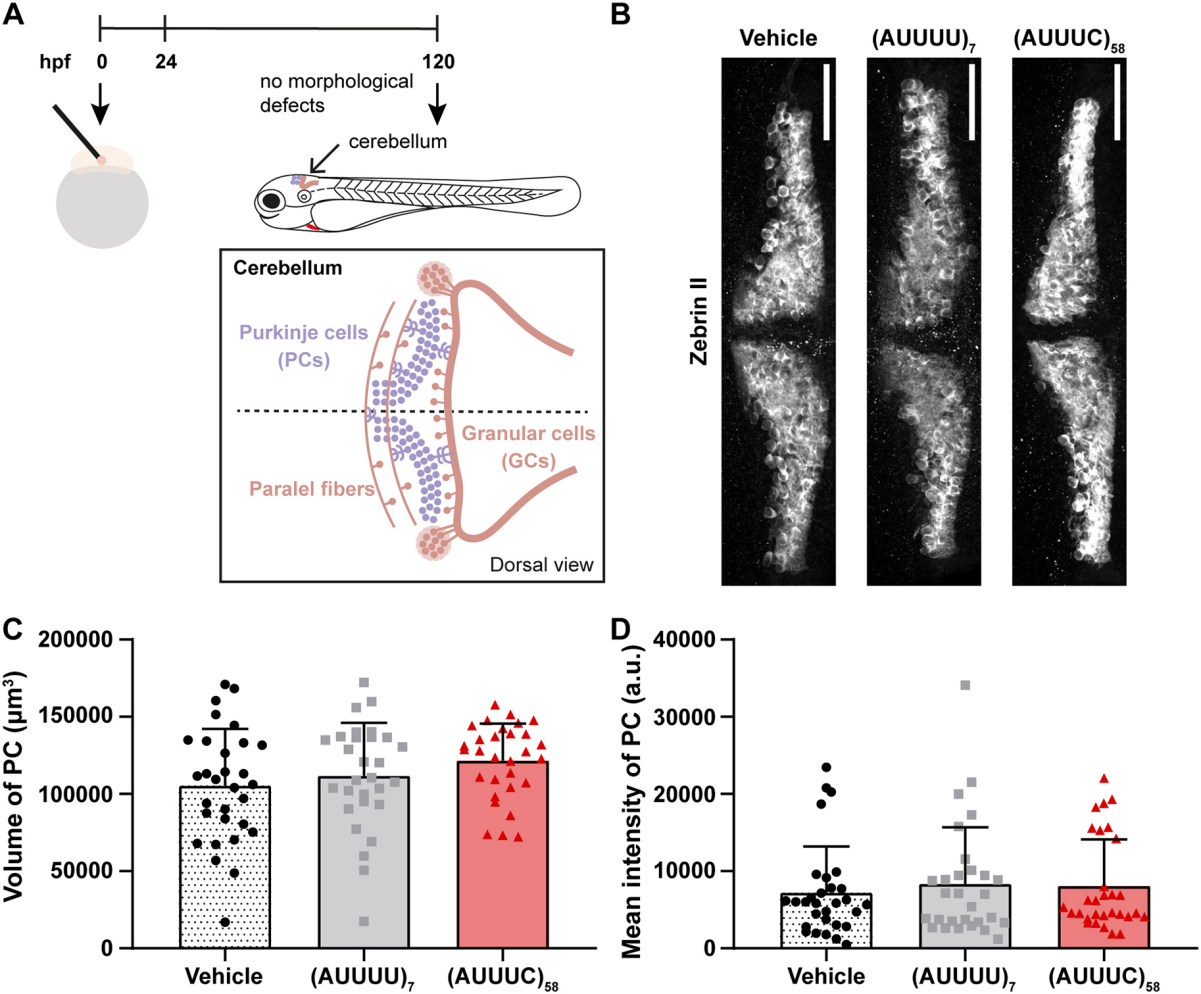
**No alterations in zebrin II-positive Purkinje cell differentiation in (AUUUC)_58_ zebrafish larvae.** (A) Schematic outlining the assessment of zebrin II-positive Purkinje cells in zebrafish larvae. One- to two-cell-stage embryos were microinjected with the non-pathogenic (AUUUU)_7_ RNA, pathogenic (AUUUC)_58_ RNAs or vehicle control, and dead or defective embryos removed at 24 hpf. Anti-zebrin II immunofluorescence was performed at 120 hpf to analyze Purkinje cells. (B) Representative z-projection images depicting zebrin II-positive Purkinje cells across conditions (scale bars: 50 µm). (C) Plot showing the volume of Purkinje cells (PCs) (one-way ANOVA). (D) Plot showing the mean intensity of Purkinje cells (Kruskal–Wallis test) as arbitrary units (a.u.). Data are presented as mean+standard deviation. Data are from three replicates including at least nine larvae per condition.

A consistent phenotype in many types of spinocerebellar ataxia is a marked reduction in the number of cerebellar Purkinje cells, which has been observed in post-mortem patient tissues (Klockgether et al., 2019). This phenotype could result from an early neurodevelopmental defect impairing Purkinje cell differentiation, as well as from late Purkinje cell degeneration due to sustained expression of the pathogenic repeat (Shabani and Hassan, 2023). Despite structural and gene expression differences, the zebrafish cerebellum – like that of mammals – is composed of a three-layer structure with similar granular, Purkinje and molecular cell layers (Bae et al., 2009). During cerebellar development in zebrafish both neural progenitors of Purkinje and granule cells start their differentiation at 72 hpf, and the three-layer structure is completed at 120 hpf (Kani et al., 2010). Because human RNA repeats have been identified to be present in the first two days after zebrafish embryo microinjection in this current study and other studies (Todd et al., 2014) and then depleted, we investigated the induction of early and late neuronal phenotypes by (AUUUC)_58_ RNA expression within this narrow embryonic time window. To test whether this transient expression can impair differentiation of Purkinje progenitor cells, we microinjected one- to two-cell-stage zebrafish embryos with the pathogenic (AUUUC)_58_ RNA, the non-pathogenic (AUUUU)_7_ RNA or vehicle. We selected and raised the microinjected embryos that did not show gross developmental malformations to analyze Purkinje cells at 120 hpf (Fig. 2A,B). We quantified the cellular volume indicated by using anti-zebrin II antibody that is known to label all Purkinje cells in zebrafish (Bae et al., 2009; Lannoo et al., 1991a,b) as a proxy for estimating the number of Purkinje cells, finding no significant differences among experimental conditions [vehicle: 105,456±36,670 µm^3^; (AUUUU)_7_: 111,705±34,446 µm^3^; (AUUUC)_58_: 121,519±24,115 µm^3^] (Fig. 2C). To scrutinize these results, we further assessed the mean intensity of the zebrin II fluorescent signal in larvae, finding no significant differences among treatments [vehicle: 7219±5961 arbitrary units (a.u.); (AUUUU)_7_: 8352±7337 a.u.; (AUUUC)_58_: 8069±6034 a.u.] (Fig. 2D). These results suggest that the transient expression of (AUUUC)_58_ RNA in early zebrafish development does not impair the differentiation of zebrin II-positive Purkinje cells.

Because cerebellar tissue of SCA37 patients shows a loss of Purkinje cells and reduced molecular layer thickness (Corral-Juan et al., 2018) where excitatory signals are sent from axons of granule cells (parallel fibers) to Purkinje cell dendrites, we also wanted to rule out the possibility that transient SCA37-associated (AUUUC)_58_ RNA expression during the first hours of development leads to these phenotypes in adulthood. With this in mind, we raised the microinjected embryos to 19 months of age and analyzed the cerebellum of adult zebrafish (Fig. S5A). We measured the cerebellar molecular cell layer area and analyzed the number of cerebellar Purkinje cells after immunostaining for zebrin II (Fig. S5B). We found no significant changes in arborization of Purkinje cells when analyzing the cerebellar molecular layer area between (AUUUC)_58_ animals and controls [vehicle: 218,761±58,368 µm^2^; (AUUUU)_7_: 193,656±40,226 µm^2^; (AUUUC)_58_: 222,280±68,435 µm^2^) (Fig. S5C). However, the total number of zebrin II-positive Purkinje cells was decreased in both the non-pathogenic (AUUUU)_7_ (35±10 cells; *P*=0.001) and pathogenic (AUUUC)_58_ animals (50±19 cells; *P*=0.02) when compared with vehicle control animals (80±13 cells) (Fig. S5D). These findings demonstrate that transient expression of SCA37-associated (AUUUC)_58_ RNA during early zebrafish development has no specific effect on the total number of Purkinje cells. Overall, these results support the idea that the cerebellar phenotypes observed in SCA37 patients, particularly those affecting Purkinje cells, are not caused by early expression of the pathogenic (AUUUC)_58_ RNA alone. Instead, they seem to be rather linked to the degeneration of cerebellar cells due to the sustained expression of the pathogenic RNA repeat.

### The SCA37-associated (AUUUC)_58_ RNA impairs axonal growth and dorsal-ventral neuromuscular innervation

**Fig. 3.**
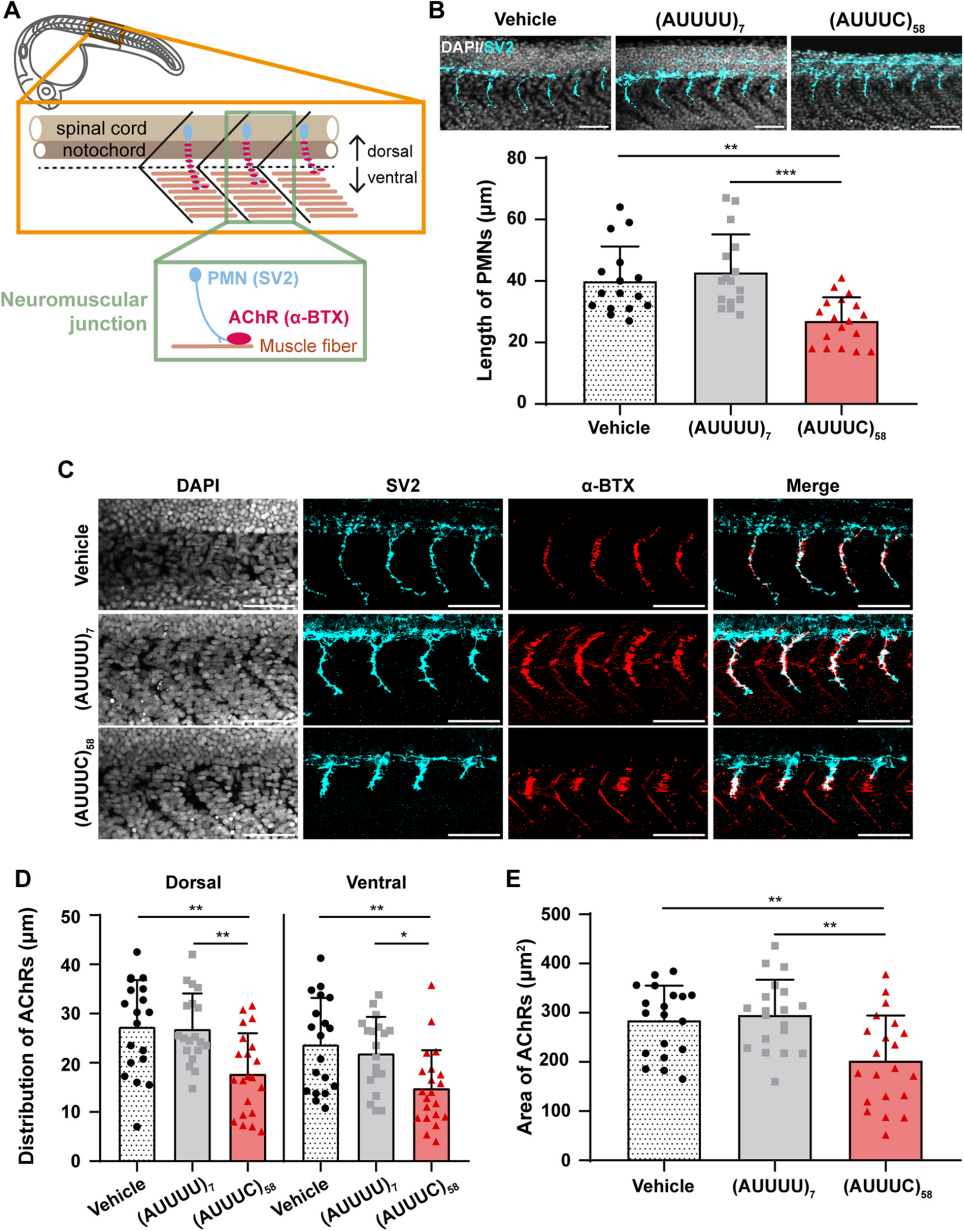
**Pathogenic (AUUUC)_58_ RNA induces developmental defects in PMN axons and AChR distribution in zebrafish muscle.** (A) Schematic of the analyzed region in zebrafish, depicting 24-hpf primary motor neuron (PMN) axons, innervating muscle cells where acetylcholine receptors (AChRs) are localized. (B) Top: Representative z-average projections of PMNs axons in embryos microinjected with vehicle, non-pathogenic (AUUUU)_7_ or pathogenic (AUUUC)_58_ RNAs in the six-somite region anterior to the cloaca (scale bars: 50 µm). Bottom: Quantification of PMN axon lengths in the described embryos [vehicle and (AUUUU)_7_: *n*=16: (AUUUC)_58_: *n*=18; three replicates; **P<0.01 and ***P<0.001, Kruskal–Wallis with Dunn's post-hoc test]. (C) Representative z-maximum projections of neuromuscular junctions (NMJs) in the four-somite-spanning region anterior to the cloaca (scale bars: 50 µm). (D) Plot showing the AChR signal distribution along the dorsal-ventral muscle axis in the above-described embryos [vehicle and (AUUUU)_7_: *n*=19 and (AUUUC)_58_: *n*=21; three replicates; *P<0.05 and **P<0.01, one-way ANOVA with Bonferroni correction]. (E) Quantification of NMJ area [vehicle and (AUUUU)_7_: *n*=19, (AUUUC)_58_: *n*=21; three replicates; **P<0.01, one-way ANOVA with Bonferroni correction]. Data are presented as the mean+standard deviation.

**Fig. 4.**
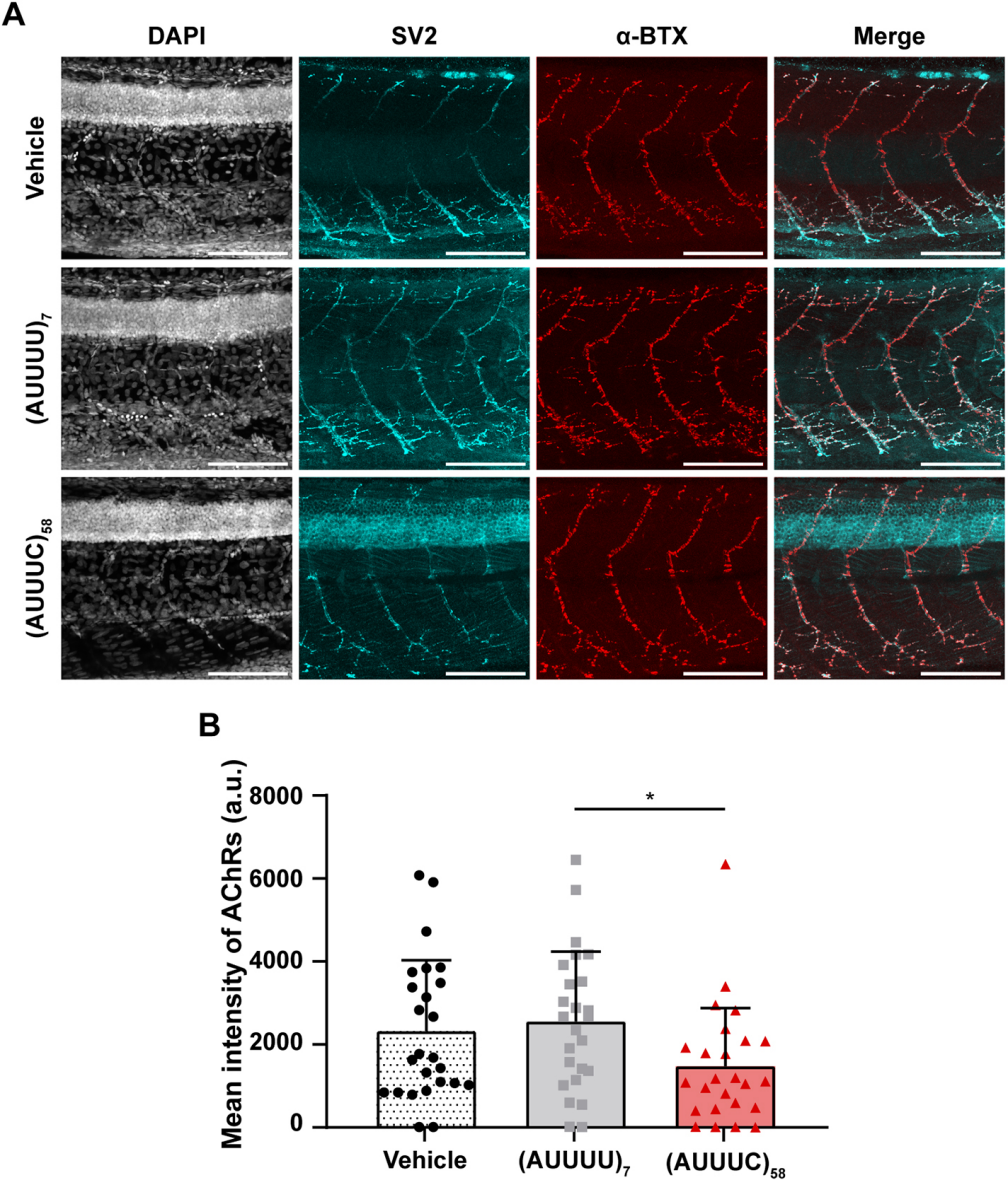
**Decreased levels of AChR clusters in the muscle of (AUUUC)_58_ zebrafish larvae at 72 hpf.** Neuromuscular junction (NMJ) analysis in zebrafish. (A) Representative z-maximum projections of primary motor neurons (PMNs) and acetylcholine receptors (AChRs) in larvae microinjected with vehicle, (AUUUU)_7_ or (AUUUC)_58_ RNAs (25 larvae per condition; four replicates each). Scale bars: 100 µm. (B) Quantification of AChRs in NMJs (as arbitrary units, a.u.) (*P<0.05, Kruskal–Wallis with Dunn's post-hoc test). Data are presented as the mean+standard deviation.

Since axonal pathology is a hallmark of SCAs (Wilson et al., 2023), and cerebellar axonal degeneration (Corral-Juan et al., 2018) and lower motor neuron impairment (Rosenbohm et al., 2022) have been found in SCA37, we investigated whether the SCA37-associated (AUUUC)_58_ RNA impairs axonal outgrowth. To test this hypothesis, we analyzed the axons of zebrafish primary motor neurons (PMNs) that exit the spinal cord and extend along each myotome segment towards ventral musculature at 24 hpf (Fig. 3A and Fig. S6A,B), by using the presynaptic anti-SV2 antibody that recognizes synaptic vesicles (Panzer et al., 2005). We measured the length of PMN axons in 24 hpf zebrafish embryos and observed significantly shorter axons in (AUUUC)_58_-injected embryos (27±8 µm) compared with embryos microinjected with the non-pathogenic (AUUUU)_7_ RNA (43±12 μm, *P*=0.0003) or vehicle control (40±11 µm, *P*=0.004) (Fig. 3B). To minimize the possibility of developmental delay in the (AUUUC)_58_-injected embryos, we compared PMN axon lengths in wild-type embryos at 19, 22 and 24 hpf (Fig. S6B,C). Indeed, we found that the (AUUUC)_58_ RNA microinjection does not impair the initiation of PMN axonal extension but rather slows down its ventral projection (Fig. S6C,D). These results show that PMN axonal outgrowth is compromised by the (AUUUC)_58_ RNA, providing evidence of disrupted synaptic innervation between spinal cord and muscle tissue. This further suggests that the (AUUUC)_58_ RNA also impairs axonal outgrowth in other neuronal cells. We also confirmed that the shortening of PMN axons is specifically induced by (AUUUC)_58_ RNA but not by large AUUUU repeat RNA *per se*, by conducting the experiment with the large non-pathogenic (AUUUU)_139_ RNA (Fig. S7). To investigate further whether the (AUUUC)_58_ RNA alters muscle innervation by PMNs and the establishment of the neuromuscular junction (NMJ), we carried out whole-mount immunofluorescence in 24 hpf embryos, labeling the presynaptic PMNs with anti-SV2 antibody and the postsynaptic acetylcholine receptors (AChRs) in muscle with tetramethylrhodamine (TRITC)-conjugated α-bungarotoxin (α-BTX) (Panzer et al., 2005) (Fig. 3C and Fig. S8A). This double staining allowed us to identify NMJs, i.e. synaptic connections between the PMN terminal ends and muscle fibers (Panzer et al., 2005). Therefore, we evaluated the colocalization of these two markers along PMN axons and found no clear differences in embryos microinjected with the pathogenic (AUUUC)_58_ RNA compared with those microinjected with the non-pathogenic (AUUUU)_7_ RNA or vehicle (Pearson's colocalization coefficient 0.3±0.1 in all conditions) (Fig. 3C and Fig. S8B). These results suggest a coupling of pre- and postsynaptic structures between PMN axons and muscle cells in all conditions, indicating that the distribution of AChRs in the muscle of (AUUUC)_58_-injected embryos is less extensive in the dorsal-ventral axis, consistent with the reduced length of PMN axons observed in these embryos. To test this hypothesis, we measured the distribution of AChRs in the dorsal and ventral regions of the embryos. Our results showed that the distribution of AChRs in muscle is reduced in both dorsal and ventral compartments in embryos microinjected with the pathogenic (AUUUC)_58_ RNA (dorsal: 18±8 µm; ventral: 15±8 µm) compared with those microinjected with the non-pathogenic (AUUUU)_7_ RNA (dorsal: 27±7 μm, *P*=0.003; ventral: 22±7 μm, *P*=0.02) or vehicle (dorsal: 27±9 µm, *P*=0.002; ventral: 24±9 µm, *P*=0.003) (Fig. 3D). To investigate potential compensatory mechanisms for the dorsal and ventral loss of AChRs, either through anterior-posterior redistribution or changes in the level of distribution, we measured the area of AChR clusters and the amount of AChRs by means of α-BTX signal or its corresponding average intensity, respectively. We observed a decrease in the area of AChR clusters [vehicle: 285±70 µm^2^; (AUUUU)_7_: 296±72 µm^2^; (AUUUC)_58_: 203±92 µm^2^; *P*=0.001, *P*=0.005] (Fig. 3E) but no alterations of their fluorescence intensity in pathogenic (AUUUC)_58_ RNA microinjected embryos compared with controls [vehicle: 335±366 a.u.; (AUUUU)_7_: 385±256 a.u.; (AUUUC)_58_: 207±189 a.u.] (Fig. S8C). These results demonstrated that the average number of AChRs by area is not different in the dorsal-ventral axis across conditions, showing no changes in terms of AChR number along the shorter axonal innervation in embryos microinjected with (AUUUC)_58_ RNA compared with controls. As NMJs are not completely established at 24 hpf (Panzer et al., 2005), we carried out whole-mount immunofluorescence with the presynaptic SV2 antibody and the postsynaptic α-BTX at 72 hpf (Fig. 4A and Fig. S9A). We detected decreased α-BTX fluorescence intensity in (AUUUC)_58_ larvae (1475±1403 a.u.) compared with (AUUUU)_7_ larvae at 72 hpf (2559±1678 a.u.; *P*=0.034) (Fig. 4B), also indicating at this stage reduced postsynaptic receptor availability and, consequently, compromised NMJ innervation. Moreover, we observed no significant differences in the dorsal-ventral distribution of AChRs across conditions [vehicle _dorsal_: 116±11 µm; (AUUUU)_7 dorsal_: 112±12 µm; (AUUUC)_58_ _dorsal_: 109±12 µm; and vehicle _ventral_: 158±12 µm; (AUUUU)_7_ _ventral_: 151±16 µm; (AUUUC)_58_ _ventral_: 155±23 µm] (Fig. S9B). As a defective distribution of AChRs was observed in the (AUUUC)_58_ embryos at 24 hpf (Fig. 3D), this suggests a normalization of AChRs distribution along the dorsal-ventral axis at 72 hpf, likely caused by RNA depletion. We confirmed that both presynaptic and postsynaptic markers colocalize also at this stage (Pearson's colocalization coefficient 0.3±0.1 in all conditions) (Fig. S9C). Overall, the transient expression of the pathogenic (AUUUC)_58_ RNA impairs axonal outgrowth, and dorsal and ventral neuromuscular innervation at 24 hpf, and leads to decreased AChRs levels in muscle at 72 hpf, suggesting that animals may develop locomotion defects.

### The pathogenic (AUUUC)_58_ RNA disrupts spontaneous early motor activity

**Fig. 5.**
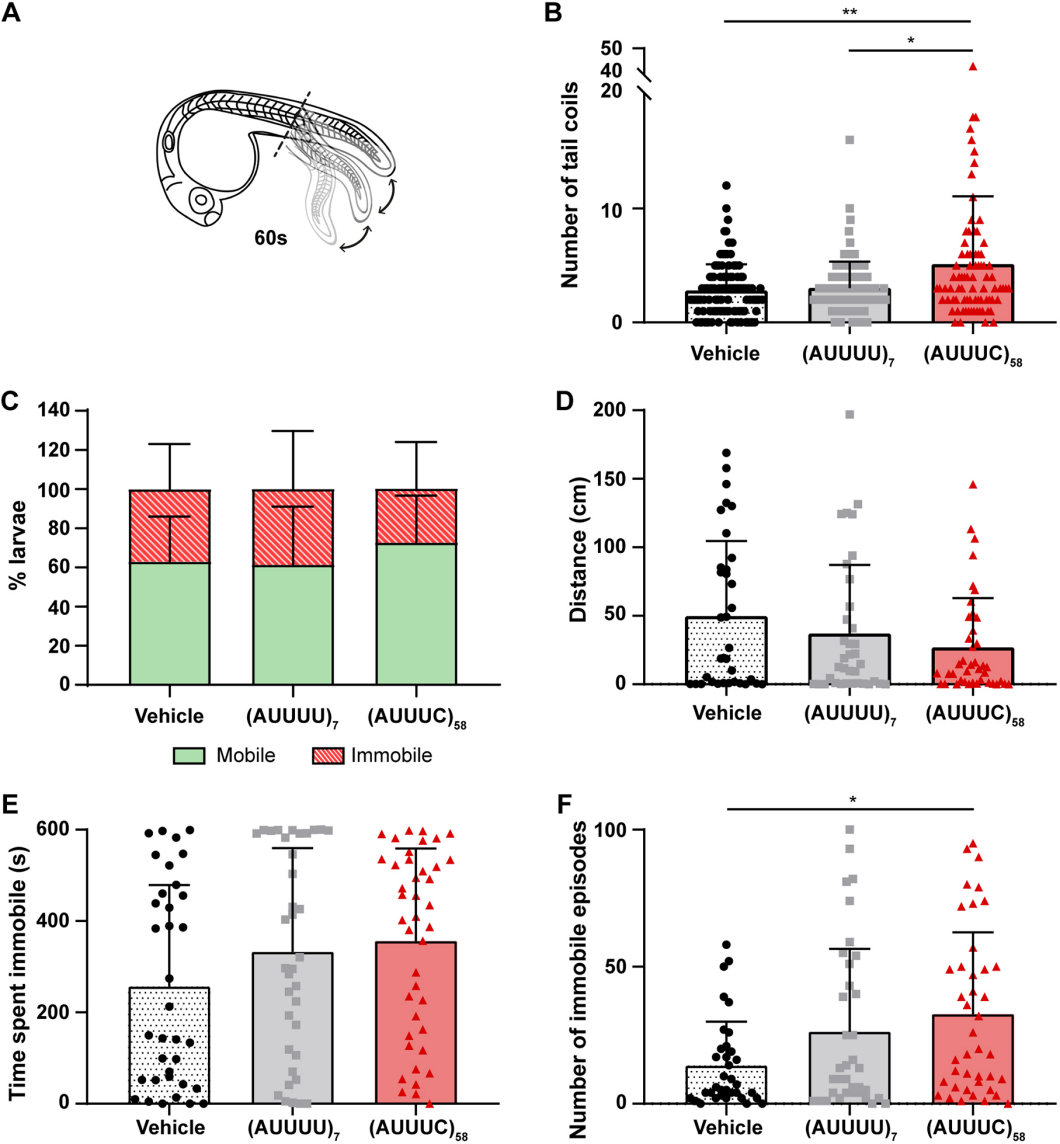
**(AUUUC)_58_-injected zebrafish exhibit hyperactive coiling behavior.** (A) Schematic of involuntary tail movement of 24 hpf zebrafish embryos. (B) Number of tail coils within 60 seconds by zebrafish embryos at 24 hpf [vehicle and (AUUUU)_7_: *n*=112, (AUUUC)_58_: *n*=82; seven replicates; *P<0.05 and **P<0.01, Kruskal–Wallis with Dunn's post-hoc test]. (C) Percentage of mobile and immobile larvae during a 10-min spontaneous locomotor test at 144 hpf [vehicle: *n*=55, (AUUUU)_7_: *n*=58, (AUUUC)_58_: *n*=57; three replicates; χ^2^ test]. (D) Total swimming distance of zebrafish larvae at 144 hpf [vehicle: *n*=35, (AUUUU)_7_: *n*=36, (AUUUC)_58_: *n*=41; three replicates; Kruskal–Wallis test]. (E) Time spent immobile by zebrafish larvae at 144 hpf [vehicle: *n*=35, (AUUUU)_7_: *n*=36, (AUUUC)_58_: *n*=41; three replicates; Kruskal–Wallis test]. (F) Number of immobility episodes in larvae at 144 hpf, i.e. immobile for ≥2 s [vehicle: *n*=35, (AUUUU)_7_: *n*=36, (AUUUC)_58_: *n*=41; three replicates; *P<0.05, Kruskal–Wallis with Dunn's post-hoc test). Data are presented as the mean+standard deviation.

Because SCA37-associated (AUUUC)_58_ RNA microinjection leads to defective axonal outgrowth of PMNs and muscle innervation, we inferred that other important neuronal defects may also be induced, leading to motor dysfunction. To test this hypothesis, we tested locomotor activity induced by a touch stimulus and spontaneous motor activity. For the former, we performed the touch-evoked escape response test in previously microinjected larvae with the (AUUUC)_58_ RNA and controls at 72 hpf (Fig. S10A). We observed no significant differences in the percentage of larvae responding to touch stimuli among groups [60% for vehicle, 57% for (AUUUU)_7_ and 59% for (AUUUC)_58_] (Fig. S10B). In larvae that responded to the touch stimulus, we analyzed the time between stimulus and response (reaction time) and the duration of the escape response (duration of the response swimming) for each larva. We found no differences in the reaction time [vehicle: 0.2±0.1 s; (AUUUU)_7_: 0.3±0.2 s; (AUUUC)_58_: 0.2±0.1 s] (Fig. S10C) or swimming duration response [vehicle: 2.3±1.8 s; (AUUUU)_7_: 2.1±1.8 s; (AUUUC)_58_: 2.0±1.7 s] (Fig. S10D) of larvae microinjected with the (AUUUC)_58_ RNA when compared with controls. These results indicate that the neuronal alterations at 24 hpf are insufficient to impair the response of larvae to a touch stimulus later at 72 hpf. Next, we tested whether the early transient presence of the pathogenic (AUUUC)_58_ RNA could affect spontaneous motor activity. Specifically, we assessed the number of spontaneous tail coils at 24 hpf and the free-swimming behavior of larvae at 144 hpf. Thus, we measured the number of spontaneous tail coils over 60 s (Fig. 5A). Spontaneous tail coiling represents the first involuntary movement in embryos and serves as a measure of synaptic communication between spinal cord and muscle cells (Drapeau et al., 2002). We observed a significant increase in tail coiling in embryos microinjected with the (AUUUC)_58_ RNA (5±6 coils) compared with those microinjected with non-pathogenic (AUUUU)_7_ RNA (3±2 coils, *P*=0.043) or vehicle controls (3±2 coils, *P*=0.003) (Fig. 5B and Movies 1-3), supporting the hypothesis that the (AUUUC)_58_ RNA alters spinal cord-muscle innervation, leading to an aberrant increase in tail movements. Apart from these very early spontaneous movements, we further analyzed the free-swimming behavior of the larvae at 144 hpf previously microinjected with the pathogenic (AUUUC)_58_ RNA, the non-pathogenic (AUUUU)_7_ RNA or the vehicle. We analyzed the percentage of immobile larvae during the 10-min test in each group, finding no significant differences among them [37% for vehicle, 39% for (AUUUU)_7_ and 27% for (AUUUC)_58_] (Fig. 5C). For motile larvae, we measured the distance swum [vehicle: 50±55 cm; (AUUUU)_7_: 37±50 cm; (AUUUC)_58_: 27±36 cm] (Fig. 5D) and the average time spent immobile for each larva during the test [vehicle: 257±222 s; (AUUUU)_7_: 333±227 s; (AUUUC)_58_: 356±202 s] (Fig. 5E) and found no significant differences across conditions. Considering an immobile episode as a 2-second interval with no locomotor activity, we found that larvae microinjected with the pathogenic (AUUUC)_58_ RNA exhibited an increased number of immobility episodes (33±30 episodes) compared with vehicle controls (14±16 episodes, *P*=0.02) (Fig. 5F). Overall, these results indicate that the transient expression of SCA37-associated (AUUUC)_58_ RNA in early zebrafish embryonic development exacerbates spontaneous locomotor activity at 24 hpf by increasing the number of tail movements but does not have a direct effect later, at 144 hpf. Our results showed that SCA37-associated (AUUUC)_58_ RNA disrupts PMN axonal outgrowth and alters neuromuscular innervation. Although these changes are unlikely to directly cause the observed spontaneous coiling phenotype (e.g. PMNs innervate fast muscle fibers that are not involved in zebrafish coiling activity) (Naganawa and Hirata, 2011), similar defects in other neuronal populations might, indeed, underlie the observed motor alterations.

### Transient expression of the SCA37-associated (AUUUC)_58_ RNA in early zebrafish development alters locomotor profile in adulthood

**Fig. 6.**
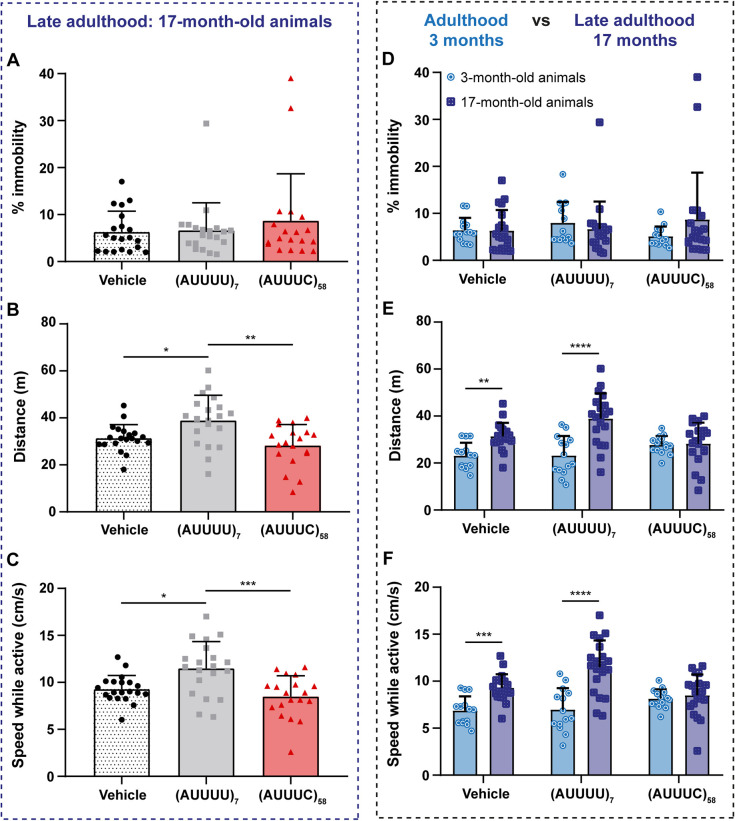
**Pathogenic (AUUUC)_58_ causes motor impairment in late-adult zebrafish at 17 months and negatively affects motor performance in fish aged 3 to 17 months.** (A-C) Novel diving tank test conducted on microinjected animals at 17 months [vehicle and (AUUUU)_7_: *n*=20, (AUUUC)_58_: *n*=19]. Percentage of immobile animals (Kruskal–Wallis test) (A). Total swimming distance (*P<0.05 and **P<0.01, two-way ANOVA with condition and sex as factors) (B). Average swimming speed (*P<0.05 and ***P<0.001, two-way ANOVA with condition and sex as factors) (C). (D-F) Novel diving tank test performed with animals aged 3 months (*n*=14 animals per condition) and 17 months [vehicle and (AUUUU)_7_: *n*=20, (AUUUC)_58_: *n*=19]. Percentage of immobile animals (Mann–Whitney *U*-test) (D). Total swimming distance (**P<0.01 and ****P<0.0001, two-way ANOVA with condition and age as factors) (E). Average swimming speed while animals are active (***P<0.001 and ****P<0.0001, two-way ANOVA with condition and age as factors) (F). Data are shown as the mean+standard deviation.

Given the alterations found during zebrafish development, we asked whether the transient expression of the SCA37-associated (AUUUC)_58_ RNA has a long-term impact on zebrafish locomotor function. Therefore, we performed the novel diving tank test at 3 months in animals previously microinjected at one- to two-cell stage with the pathogenic (AUUUC)_58_ RNA, non-pathogenic (AUUUU)_7_ RNA, or vehicle. This test consists of placing the zebrafish into a new tank and recording its movements for 6 min, allowing the analysis of animal locomotor activity. The 3-month-old animals did not show significant differences in the percentage of time spent immobile [vehicle: 6±3%; (AUUUU)_7_: 8±4%; (AUUUC)_58_: 5±2%] (Fig. S11A), in the distance swum [vehicle: 23±6 m; (AUUUU)_7_: 23±8 m; (AUUUC)_58_: 28±4 m] (Fig. S11B) or displacement velocity compared with control animals [vehicle: 7±2 cm/s; (AUUUU)_7_: 7±2 cm/s; (AUUUC)_58_: 8±1 cm/s] (Fig. S11C). We did not observe any significant interaction between the factors condition and sex in the observed locomotor activity (Fig. S11). Additionally, no differences in body length and weight of animals were found among experimental groups [vehicle: 1.95±0.25 cm and 126±94 mg; (AUUUU)_7_: 1.98±0.34 cm and 129±64 mg; (AUUUC)_58_: 2.12±0.23 cm and 166±87 mg] (Fig. S12A,B). Given that SCA37 is a late-onset disease (Seixas et al., 2017), we performed the novel diving tank test on 17-month-old zebrafish to investigate locomotor dysfunction in late adults. While no significant differences were observed in the percentage of time spent immobile across conditions [vehicle: 6±4%; (AUUUU)_7_: 7±6%; (AUUUC)_58_: 9±10%] (Fig. 6A), zebrafish microinjected with pathogenic (AUUUC)_58_ RNA exhibited a significant shorter swimming distance (28±9 m) and reduced speed (9±2 cm/s) compared with those microinjected with non-pathogenic (AUUUU)_7_ RNA (39±11 m, *P*=0.001 and 12±3 cm/s, *P*=0.0005, respectively) (Fig. 6B,C), demonstrating a locomotor dysfunction in these animals. Furthermore, we observed no differences in the length and weight across conditions [vehicle: 2.86±0.19 cm and 585±184 mg; (AUUUU)_7_: 2.95±0.16 cm and 533±113 mg; (AUUUC)_58_: 2.89±0.22 cm and 564±206 mg] (Fig. S12C,D). Similar to the results obtained with 3-month-old animals, no interaction was observed between condition and sex, suggesting no sex-specific differences in the locomotor profile of the animals at 17 months of age (Fig. 6A-C). When comparing the locomotor performance of 3- and 17-month-old adult zebrafish, no significant differences were observed in the percentage of time spent immobile [vehicle: t_3mth_=6±3% and t_17mth_=6±4%; (AUUUU)_7_: t_3mth_=8±4% and t_17mth_=7±6%; (AUUUC)_58_: t_3mth_=5±2% and t_17mth_=9±10%] (Fig. 6D). However, in comparison, 17-month-old control animals swam farther [vehicle: d_3mth_=23±6 m and d_17mth_=31±6 m, *P*=0.003; (AUUUU)_7_: d_3mth_=23±8 m and d_17mth_=39±11 m, *P*<0.0001] (Fig. 6E) and faster [vehicle: v_3mth_=7±2 cm/s and v_17mth_=9±1 cm/s, *P*=0.001; (AUUUU)_7_: v_3mth_=7±2 cm/s and v_17mth_=12±3 cm/s, *P*<0.0001] (Fig. 6F) than the 3-month-old control animals. This increase in performance was expected, as animals typically grow (length and weight) between 3 and 17 months of age (see Fig. S12). Although zebrafish microinjected with pathogenic (AUUUC)_58_ RNA exhibited an increase in size as control conditions (Fig. S12), they did not show the corresponding increase in locomotor performance (d_3mth_=28±4 m and d_17mth_=28±9 m; v_3mth_=8±1 cm/s and v_17mth_=8±2 cm/s) (Fig. 6E,F). Thus, there was a significant interaction when factoring in microinjected RNA and age (*P*=0.0008 for distance and *P*=0.0004 for speed), which shows that these animals experience locomotor impairment that becomes evident in late adulthood.

### NOVA2 rescues defective axonal outgrowth in zebrafish with the pathogenic (AUUUC)_58_ RNA

**Fig. 7.**
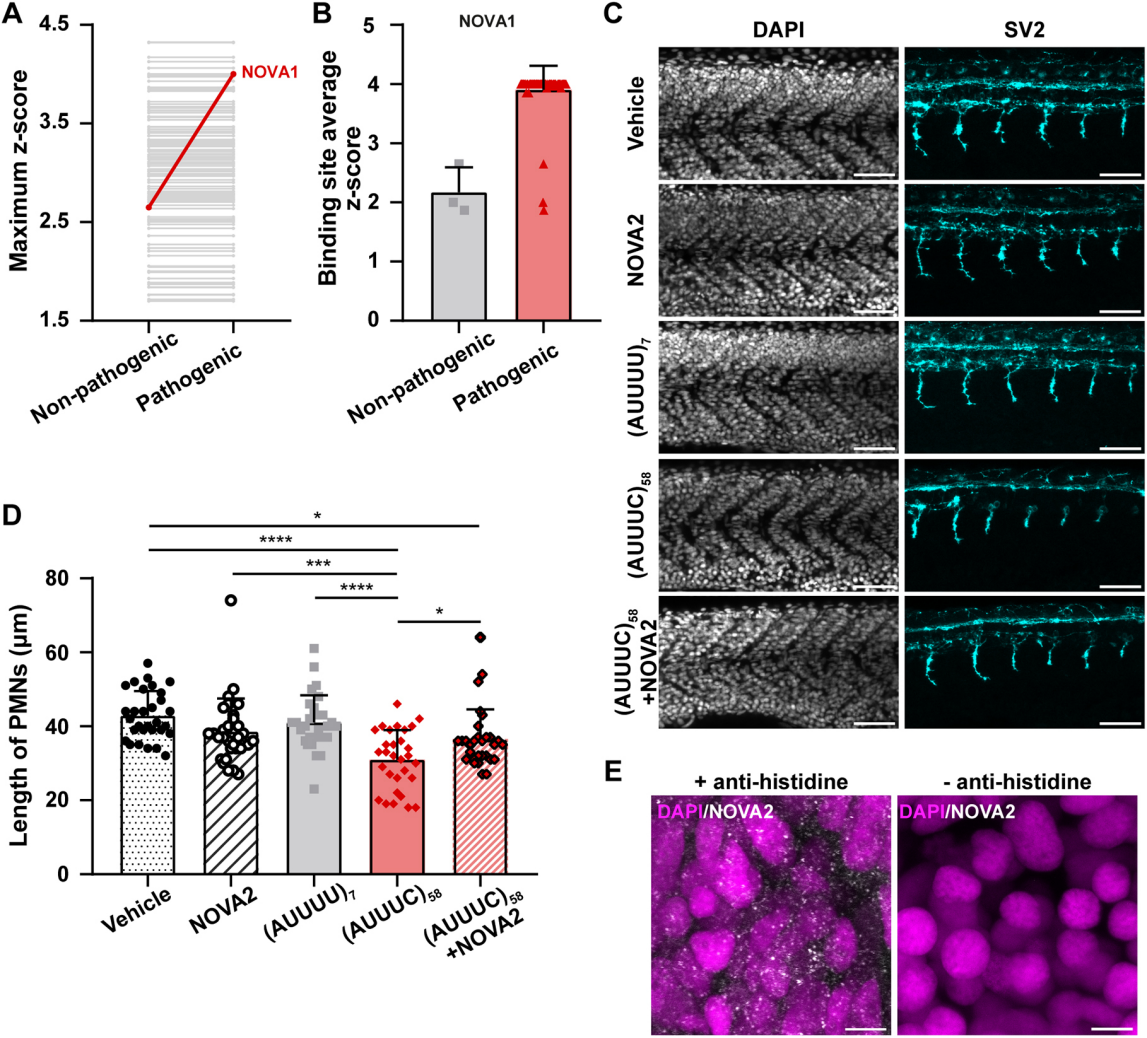
**NOVA2 mitigates axonal defects caused by pathogenic (AUUUC)_58_ RNA in zebrafish PMNs.** (A) Maximum z-scores of putative RNA-binding proteins (RBPs) that bind non-pathogenic (AUUUU)_199_ or pathogenic (AUUUC)_58_ RNA, assessed by using the RBPmap tool (*P*<0.05). (B) Average z-score for each binding site for NOVA1 within (AUUUU)_199_ or (AUUUC)_58_ RNA. Data are shown as the mean+standard deviation. (C) Representative z-average projections of zebrafish primary motor neuron (PMN) axons in the six-somite region anterior to the cloaca at 24 hpf (scale bars: 50 µm). (D) Length of PMN axons in embryos microinjected with either vehicle, NOVA2 (*NOVA2*), (AUUUU)_7_, (AUUUC)_58_, or a combination of (AUUUC)_58_ and *NOVA2* [(AUUUC)_58_ +NOVA2] mRNA (*n*=30 embryos per condition; three replicates; *P<0.05, ***P<0.001 and ****P<0.0001, two-way ANOVA with conditions and replicates as factors, followed by Bonferroni post-hoc test for axon length, after applying a cube root data transformation). Raw data are represented as the mean+standard deviation. (E) Z-projection images showing the nuclear and cytoplasmic distribution of human NOVA2 in 24 hpf zebrafish muscle after *NOVA2* mRNA microinjection. Representative z-maximum projections with (left) and without (right) anti-histidine primary antibody (white). Nuclei were stained with DAPI (purple). Scale bars: 5 µm.

One of the mechanisms associated with the pathogenic (AUUUC)_58_ RNA in SCA37 was expected to involve sequestration of RBPs, leading to their functional impairment. Therefore, we performed an unbiased *in silico* analysis to identify RBPs with high binding affinity to the pathogenic (AUUUC)_58_ RNA by using the RBPmap webserver (Paz et al., 2014). We found 109 putative RBPs predicted to bind to the non-pathogenic (AUUUU)_199_ and pathogenic (AUUUC)_58_ RNAs (*P*<0.05; Table S1). Next, we determined the maximum z-score for each RBP hit, detecting NOVA1 as the unique RBP in this database, which binds with higher affinity to pathogenic (AUUUC)_58_ RNA than non-pathogenic RNA (Fig. 7A). We also quantified the number of binding sites for NOVA1 in both non-pathogenic and SCA37-associated (AUUUC)_58_ RNAs. We found 61 binding sites in (AUUUC)_58_ RNA, against the three binding sites in (AUUUU)_199_ RNA, which has the same total repeat length as pathogenic RNA with flanking AUUUU repeats (Fig. 7B). Unlike NOVA1, NOVA2 RBP, which is also a neuron-specific splicing factor that binds to the same YCAY-binding motif (Saito et al., 2016) present in the AUUUC repeat itself, is not present in the RBPmap database. However, NOVA2 plays a role in axonal pathfinding (Saito et al., 2016) and is expressed in the same neuronal cells as DAB1 (Yano et al., 2010). Importantly, NOVA2 binds to the (AUUUC)_58_ RNA *in vitro* and colocalizes with (AUUUC)_58_ RNA aggregates in human neural stem cells (Figueiredo et al., 2025 preprint). Therefore, we investigated whether NOVA2 loss-of-function might be implicated in the PMN axonal defects detected in zebrafish embryos microinjected with the pathogenic (AUUUC)_58_ RNA. Given the identical NOVA2-binding motif across vertebrates (Jelen et al., 2007), we co-microinjected the pathogenic (AUUUC)_58_ RNA and the human *NOVA2* mRNA into zebrafish embryos to analyze PMN axon length at 24 hpf using whole-mount immunofluorescence with anti-SV2 antibody (Fig. 7C). We confirmed that *NOVA2* mRNA microinjection alone does not impact the length of PMN axons in zebrafish when compared with vehicle control embryos (vehicle: 43±7 µm; *NOVA2*: 39±9 µm) (Fig. 7D) after its translation into NOVA2 protein assessed by whole-mount immunofluorescence with anti-histidine antibody targeting the NOVA2 histidine tail (Fig. 7E). As previously observed, we also confirmed that the pathogenic (AUUUC)_58_ RNA microinjection leads to axonal shortening of PMNs (31±8 µm) when compared with control conditions [vehicle: 43±7 µm; (AUUUU)_7_: 41±7 µm; *P*<0.0001 for both controls] (Fig. 7D). Importantly, we found that NOVA2 suppresses the axonal shortening of PMNs triggered by the pathogenic (AUUUC)_58_ RNA, thereby rescuing the length of zebrafish PMN axons when co-microinjected with the (AUUUC)_58_ RNA [(AUUUC)_58_: 31±8 µm; (AUUUC)_58_+*NOVA2*: 37±8 µm, *P*=0.01] (Fig. 7C,D). Although embryos co-microinjected with (AUUUC)_58_ RNA and *NOVA2* mRNA exhibited shorter PMN axons [(AUUUC)_58_+*NOVA2*: 37±8 µm] compared with vehicle control embryos (vehicle: 43±7 µm), their axonal lengths were not significantly different from those observed for the other non-pathogenic conditions [*NOVA2*: 39±9 µm; (AUUUU)_7_: 41±7 µm] (Fig. 7D). These results indicate that the SCA37 pathogenic mechanism triggers a partial NOVA2 loss-of-function.

**Fig. 8.**
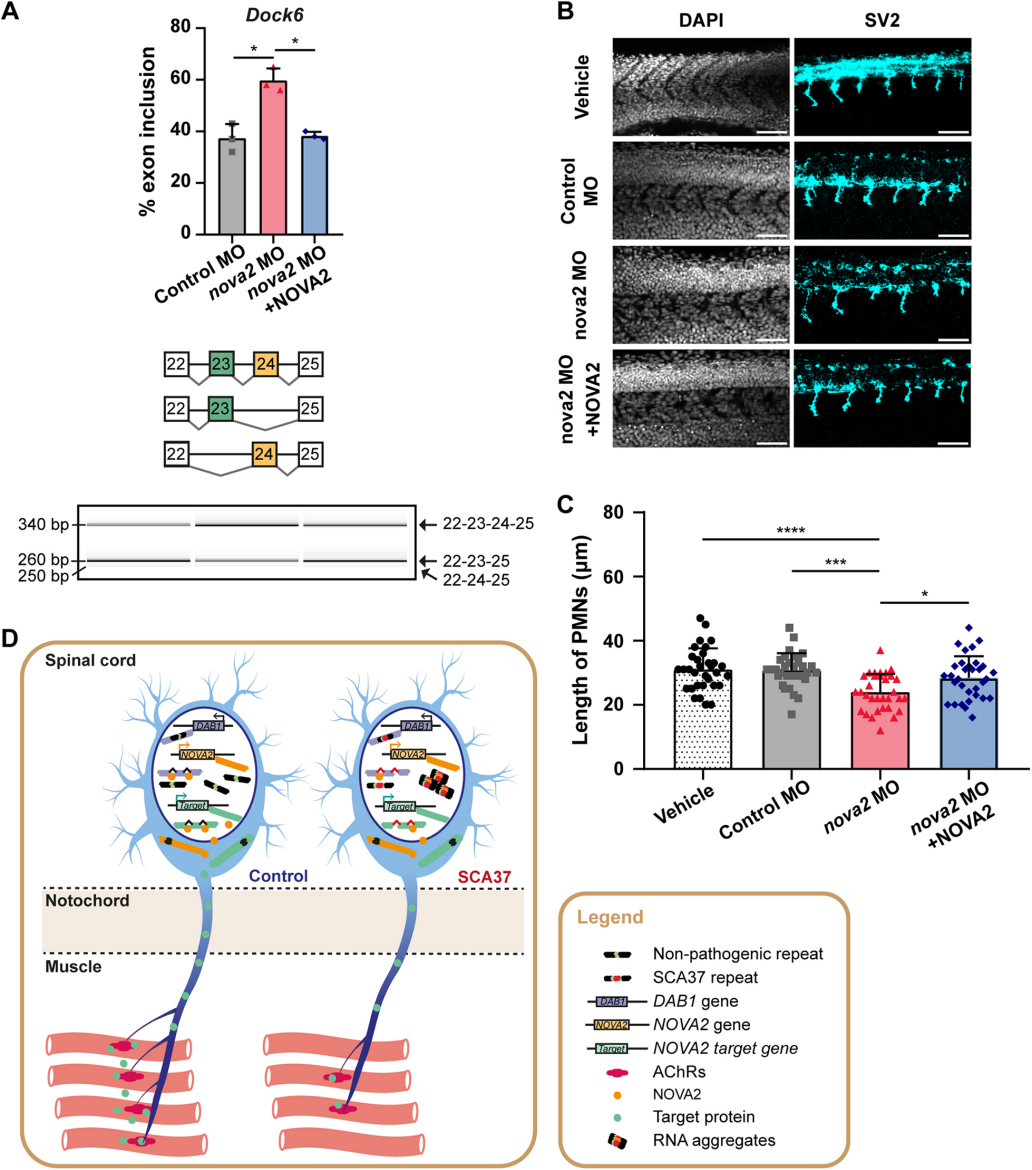
**Nova2 knockdown in zebrafish induces PMN axonopathy, recapitulating the (AUUUC)_58_ RNA microinjection effect.** (A) Top: Percentage of inclusion of exon 23 and 24 in *Dock6* at 24 hpf after microinjection of control (Control MO) or *nova2* morpholino (*nova2* MO), or co-microinjection of *nova2* morpholino and human *NOVA2* mRNA (*nova2* MO +NOVA2; (*P<0.05, one-way ANOVA with Welch correction and Dunnett's T3 post-hoc test; three biological replicates). Data are represented as mean+standard deviation. Bottom: Schematic of inclusion of alternatively spliced exons 23 and 24 in *Dock6* transcripts, along with the corresponding Bioanalyzer electrophoresis. (B) Representative z-average projections of primary motor neuron (PMN) axons in the six-somite region anterior to the cloaca in zebrafish at 24 hpf (scale bars: 50 µm). (C) Quantification of PMN axon length in embryos microinjected with vehicle, standard control morpholino (Control MO), *nova2* morpholino (*nova2* MO), or a combination of *nova2* morpholino and human *NOVA2* (*nova2* MO +NOVA2) mRNA (*n*=32 embryos per condition; three replicates; *P<0.05, ***P<0.001 and ****P<0.0001, one-way ANOVA with Bonferroni post-hoc test). Data are represented as the mean+standard deviation. (D) Hypothetical model describing (AUUUC)_58_-driven SCA37 neurodevelopmental axonopathy in zebrafish. Left: During development, *DAB1* comprising the non-pathogenic repeat is expressed in motor neurons, allowing neuromuscular junction (NMJ) formation via axonal growth towards pre-patterned clusters of acetylcholine receptor (AChR). In these neurons, NOVA2 regulates the alternative splicing of axon pathfinding-related transcripts (e.g. neuron-specific Z+ agrin protein isoforms are released along muscle tissue, stabilizing AChR clusters). Right: In SCA37 motor neurons, *DAB1* comprising the pathogenic ATTTC repeat might form (AUUUC)_58_ RNA aggregates, sequestering RNA-binding proteins (RBPs) like NOVA2. This NOVA2 loss-of-function might lead to aberrant splicing in PMNs, resulting in axonal shortening and abnormal AChR clustering/positioning near the horizontal myoseptum.

To further investigate whether *nova2* loss-of-function in zebrafish recapitulates the PMN axonal phenotype caused by the (AUUUC)_58_ RNA, we knocked down Nova2 by microinjecting a morpholino targeting the *nova2* start codon that blocks Nova2 translation (Giampietro et al., 2015). We used the same *nova2* morpholino reported by Giampietro and colleagues, following their protocol for validation by analysis of alternative splicing of Nova2 target pre-mRNAs (Giampietro et al., 2015) but, instead, used human *NOVA2* mRNA to rescue. Briefly, to validate the Nova2 knockdown upon zebrafish embryo microinjection, we extracted RNA from pooled embryos previously microinjected with either control or *nova2*-targeting morpholino at 24 hpf and analyzed Nova2-regulated alternative splicing events by reverse transcription (RT)-PCR. We quantified the percentage of alternative exon inclusions into transcripts of Nova2 targets (Giampietro et al., 2015), including transcripts encoding the cytoskeleton proteins Dock6, Rap1Gap and DBS that are, respectively, involved in neuronal processes, such as neurite outgrowth (Miyamoto et al., 2007), neuronal migration (Jossin and Cooper, 2011) and stabilization of synapses (Hayashi et al., 2013). To confirm the Nova2 loss-of-function triggered by the *nova2* morpholino, we found a significantly increased inclusion of exons 23 and 24 in *Dock6* in embryos microinjected with this morpholino compared with control morpholino-injected embryos (control MO: 37±6%; *nova2* MO: 60±5%; *P*=0.016) (Fig. 8A), as expected. Importantly, this was partially rescued by co-microinjection of the human *NOVA2* mRNA (*nova2* MO: 60±5%; *nova2* MO+NOVA2: 38±2%; *P*=0.023) (Fig. 8A), suggesting some degree of conserved NOVA2 function between zebrafish and humans. We also observed decreased inclusion of exon 18 in *Rap1Gap* between *nova2*- and control morpholino-injected embryos (control MO: 57±1%; *nova2* MO: 40±4%; *P*=0.0004) (Fig. S13A), as anticipated. Moreover, we detected a reduction in inclusion of exons 26 and 27 in *DBS* (*MCF2L*) in *nova2* morpholino-injected embryos (*nova2* MO: 0±1%), when compared with the control condition (control MO: 20±5%; *P*=0.035) (Fig. S13B), indicating Nova2 loss-of-function, according to the study used for our validation (Giampietro et al., 2015). Additionally, we investigated alternative splicing of zebrafish *Neo1* (Jelen et al., 2007) encoding a protein involved in axon outgrowth and pathfinding (Mattioli et al., 2020), which is abnormally spliced in Nova2 knockout mice (Saito et al., 2016). We also observed increased inclusion of exon 26 in *Neo1* when comparing embryos microinjected with *nova2* and control morpholinos (control MO: 99±1%; *nova2* MO: 100±0%; *P*=0.046) (Fig. S13C), as expected. These alternatively spliced transcript isoforms were confirmed by sequencing. In contrast to *Dock6* (Fig. 8A), co-microinjection of human *NOVA2* mRNA did not rescue the splicing defects induced by *nova2*-morpholino in *Rap1Gap*, *DBS* (*MCF2L*) and *Neo1* (Fig. S13), indicating that, despite their similarities, zebrafish Nova2 and human NOVA2 are not functionally equivalent. Nevertheless, the aberrantly spliced isoforms found in embryos microinjected with *nova2*-targeting morpholino, compared with control morpholino, validated the knockdown of Nova2 in zebrafish.

Next, we measured the length of PMN axons labeled with anti-SV2 antibody at 24 hpf (Fig. 8B). We detected shorter PMN axons in embryos microinjected with the *nova2*-targeting morpholino (*nova2* MO: 24±6 µm) compared with embryos microinjected with vehicle (31±7 μm; *P*=0.00006) or control morpholino (31±5 µm; *P*=0.0001) (Fig. 8C), showing that Nova2 knockdown leads to neuronal phenotypes similar to those induced by the (AUUUC)_58_ RNA. Notably, this defective axon outgrowth was partially restored by co-microinjection with human *NOVA2* mRNA (*nova2* MO: 24±6 µm; *nova2* MO+*NOVA2*: 28±7 µm; *P*=0.03) (Fig. 8C), as observed after co-microinjection of (AUUUC)_58_ RNA and human *NOVA2* mRNA in zebrafish. These results endorse a role for NOVA2 loss-of-function in SCA37.

## DISCUSSION

In this study, we demonstrated that, in zebrafish, pathogenic (AUUUC)_58_ RNA causes axon outgrowth defects in presynaptic PMNs and aberrant postsynaptic localization of AChR clusters during embryonic development. We transiently expressed the SCA37-associated (AUUUC)_58_ RNA in zebrafish embryos and demonstrated that the developmental defects caused by this RNA contribute to impaired motor function in adult animals. Furthermore, we showed that the human NOVA2 significantly rescues the axon outgrowth defects caused by (AUUUC)_58_ RNA. Thus, we propose that (1) SCA37 may be partially driven by neurodevelopment defects caused by the early expression of pathogenic (AUUUC)_58_ RNA; (2) these neurodevelopmental defects may contribute to locomotor impairment in late adulthood and; (3) a key mechanism underlying these neurodevelopmental defects is the partial loss-of-function of NOVA2, which we hypothesize to be sequestered by the pathogenic (AUUUC)_58_ RNA that contains abnormally high numbers of predicted binding sites for this RBP (Fig. 8D).

### The impact of the pathogenic AUUUC repeat RNA beyond SCA37

Pentanucleotide repeat insertions have been described to cause several neurological diseases, such as SCA10, SCA37 and FAMEs (Corbett et al., 2019; Florian et al., 2019; Ishiura et al., 2018; Morato Torres et al., 2022; Seixas et al., 2017; Yeetong et al., 2024, 2019). All these diseases are caused by the same or similar repeat motifs and related RNA-mediated mechanisms (Ishiura and Tsuji, 2020; Loureiro et al., 2022; Zhang and Ashizawa, 2022). Like SCA37, the seven types of FAME known so far are caused by an ATTTC repeat motif, and mainly characterized by cortical myoclonic tremors and sporadic tonic-clonic seizures (Corbett et al., 2019; Florian et al., 2019; Ishiura et al., 2018; Yeetong et al., 2024, 2019). Notably, there are genes, namely *CACNA1A*, that are simultaneously associated with progressive spinocerebellar ataxia and epilepsy (Alonso et al., 2003, 2004; Stam et al., 2008). The increased spontaneous coiling in zebrafish after (AUUUC)_58_ RNA microinjection might be reminiscent of abnormal excitability and warrants future investigation. Importantly, RNA foci have been detected in cortical and Purkinje cell neurons in post-mortem tissue from FAME1 patients (Ishiura et al., 2018), endorsing a common pathogenic mechanism mediated by (AUUUC)_n_ RNA in FAMEs and SCA37. Together, these observations suggest that AUUUC repeat RNA may exert pathogenic effects during neurodevelopment, contributing to disease mechanisms shared between SCA37 and FAMEs, and extending the relevance of (AUUUC)_n_-mediated toxicity beyond SCA37.

### The SCA37-associated AUUUC repeat RNA causes neurodevelopmental defects

Emerging data from human fetuses support findings that expression of pathological expanded repeats during embryonic development disrupts neural cell progenitor differentiation and neuronal connectivity, setting the stage for adult-onset neurodegeneration (Barnat et al., 2020; Hickman et al., 2021; Shabani and Hassan, 2023). Since neurodevelopment is key for the establishment of neuronal circuits and the SCA37-associated ATTTC repeat is highly expressed as a *DAB1* intronic region during embryonic stages in humans (Seixas et al., 2017), the (AUUUC)_58_ RNA has the potential to induce phenotypes in human fetuses. We showed in this current work that SCA37-associated (AUUUC)_58_ RNA in zebrafish embryos delayed the hatching process, causing severe morphological abnormalities. Even though many severely affected human infants with other types of SCA have been reported (Babovic-Vuksanovic et al., 1998; Miyazaki et al., 1996), for now they have not been examined in families with members diagnosed with SCA37 (Corral-Juan et al., 2018; Rosenbohm et al., 2022; Seixas et al., 2017). Consequently, this phenotype is likely an exacerbation of the ubiquitous pathogenic RNA overexpression, as observed for expanded myotonic dystrophy type 1 (DM1)-associated CUG repeat in microinjected zebrafish embryos (Todd et al., 2014).

Expression of (AUUUC)_58_ RNA during the first hours of development was not sufficient to cause loss of Purkinje cells in zebrafish, as also described previously in two SCA37 patients post mortem (Corral-Juan et al., 2018). Differentiation of Purkinje cells was not significantly affected at 120 hpf, probably because Purkinje cell differentiation in zebrafish starts at 72 hpf (Kani et al., 2010) and the number of (AUUUC)_58_ RNA copies is likely low due to progressive RNA degradation in the first 24 hpf. In adult zebrafish, despite the apparently similar Purkinje cell arborization across conditions, the decreased number of zebrin II-positive Purkinje cells in animals microinjected with both non-pathogenic and pathogenic RNA repeats endorses that, in zebrafish, (AUUUC)_58_ RNA is very transient to impact these cells. In contrast, human SCA37 patients exhibit increased expression of ATTTC repeat insertion in late stages of the disease (Corral-Juan et al., 2018). This is supported by the *DAB1* upregulation found in patient-derived neurons (Loureiro et al., 2026), which has not been covered in our RNA microinjection model, as it was specifically designed to investigate the relevance of neurodevelopmental RNA repeat expression for the disease. Notably, dysregulation of zebrin II expression has been reported for polyglutamine repeat diseases in mice as well as patients with motor dysfunction (Bartelt et al., 2024). However, unlike in humans, zebrin II-negative Purkinje cells have not been documented in zebrafish cerebellum (Bae et al., 2009).

Axon pathology is a common feature in neurodegenerative diseases, including SCAs, preceding cerebellar cell loss (Wilson et al., 2023). Motor neuron involvement with muscle atrophy is present in several SCAs, namely SCA2, SCA3 and SCA36 (García-Murias et al., 2012; Ghahremani Nezhad et al., 2021; Ikeda et al., 2012; Kanai et al., 2003; Paulson, 2012). The defective axonal outgrowth and muscle cell innervation observed in embryos reflect the disruptive role of the AUUUC repeat RNA in peripheral axons. Similarly, PMN axonal guidance defects in embryos of zebrafish modeling SCA13 and chromosome 9 open reading frame 72 (C9orf72)-associated frontotemporal dementia/amyotrophic lateral sclerosis (FTD/ALS) recapitulate neuronal axonopathy (Issa et al., 2012; Swinnen et al., 2018). Developmental synaptic abnormalities in the SCA1 mouse model, evidenced by defective climbing fiber innervation of cerebellar Purkinje cells (Ebner et al., 2013), lead to adult-onset motor deficits, further supported by the expression of SCA1-associated CAG repeats at specific developmental stages (Serra et al., 2006; Zu et al., 2004).

### Embryonic SCA37-associated (AUUUC)_58_ RNA drives motor dysfunction in adult zebrafish

Aberrant neural circuits have been described for SCAs (Edamakanti et al., 2018; Hsieh et al., 2020; Issa et al., 2012). Our analysis of the NMJ in (AUUUC)_58_ RNA-microinjected embryos showed abnormal innervation of muscle cells, triggered by defective motor neuron outgrowth. A similar abnormal innervation pattern is expected for other neurons in the nervous system of these animals. Because precise neuronal circuits must be established early during embryonic development, failure to form proper connections is expected to cause functional impairment. In fact, adult zebrafish that had been microinjected at embryonic stage with SCA37-associated (AUUUC)_58_ RNA presented motor dysfunction, indicating that embryonic changes in synaptic connectivity can contribute to an adult-onset motor phenotype.

### The SCA37-associated AUUUC repeat partially triggers NOVA2 loss-of-function

Transient expression of DM1- and FTD/ALS-associated expanded repeat RNAs in zebrafish produces developmental phenotypes that result in RNA-mediated pathogenesis due to RBP sequestration in these late-onset neuromuscular and neurodegenerative disorders (Swinnen et al., 2018; Todd et al., 2014). Coexpression of human *NOVA2* mRNA with (AUUUC)_58_ RNA partially rescued axonal outgrowth defects in embryos, indicating a role for NOVA2 loss-of-function in SCA37. However, this rescue – which needs future investigation regarding motor phenotype in adult zebrafish – was performed in PMNs only at 24 hpf, and axon lengths did not completely return to control levels after coexpression of human NOVA2. On the other hand, the Nova2 knockdown also demonstrated defective PMN axon outgrowth in zebrafish embryos, recapitulating the phenotype triggered by the (AUUUC)_58_ RNA. Consistent with this, another morpholino-injection-induced knockdown of zebrafish Nova2 also impaired axon outgrowth by decreasing the number of inter-tecta axonal tracts (Mattioli et al., 2020), supporting our results. In mice, loss-of-function of *Nova2* has also shown defective ventral diaphragm innervation by motor neurons (Saito et al., 2016). Altogether, this strongly suggests that the neurodevelopmental defects are mediated by impaired function of RBPs. Indeed, SCA37-associated (ATTTC)_n_ expression in human cells has shown nuclear RNA aggregation (Seixas et al., 2017). Therefore, we hypothesize that (AUUUC)_58_ RNA aggregates are at the core of the neuronal axon pathology seen in our zebrafish embryos. In support of this, NOVA2 regulates the alternative splicing of 540 genes in mice, including that of *Dab1* (Yano et al., 2010). Moreover, an abnormally spliced fetal isoform of *DAB1* has been found in cerebellar tissue of elderly SCA37 patients but not in unaffected age-matched control individuals (Corral-Juan et al., 2018). This led the authors to propose embryonic effects of the pathogenic (ATTTC)_n_ insertion to be an early trigger of SCA37 (Corral-Juan et al., 2018), consistent with this work. In addition, NOVA2 – a key player in synapse formation at the NMJ – also regulates the alternative splicing of axon guidance-related genes in mice (Saito et al., 2016). For instance, it regulates the levels of *Z+ agrin* neuron-specific isoforms released by motor neurons to control the clustering of AChRs in muscle (Ruggiu et al., 2009), among other processes. This evidence further supports the importance of NOVA2 loss-of-function in the synaptic defects triggered by SCA37-associated AUUUC repeat RNA (Fig. 8D).

In conclusion, embryonic SCA37-associated (AUUUC)_58_ RNA causes presynaptic axonopathy and impaired NMJ innervation during development, leading to adult-onset motor dysfunction. This axonopathy is partially rescued by the RBP NOVA2, pointing to its role in SCA37. This knowledge will contribute to delineate approaches for timely therapeutic intervention, with significance far beyond SCA37.

## MATERIALS AND METHODS

### Animal husbandry

Zebrafish (*Danio rerio*) husbandry and procedures were implemented following the National guidelines and legislation for housing and care of laboratory animals (Decree-Law No. 113/2013) and the European Union legislation on the protection of animals used for scientific purposes (Directive 2010/63/EU), being authorized by the i3S animal ethics committee (i.e. animal welfare organization Órgãos Responsáveis pelo Bem-Estar dos Animais (ORBEA), ref. 2018-29) and the Direção-Geral de Alimentação e Veterinária (DGAV; Ref. 022872/2020-12-31). Maintenance and handling of zebrafish animals were performed in the i3S zebrafish animal facility, which is licensed by DGAV and part of the Association for Assessment and Accreditation of Laboratory Animal Care (AAALAC) International-accredited animal care and use program.

### *In vitro* RNA synthesis

The non-pathogenic alleles comprising (ATTTT)_7_ – one of the most prevalent alleles among the non-affected population – and (ATTTT)_139_, as well as the pathogenic allele with the (ATTTT)_57_(ATTTC)_58_(ATTTT)_84_ insertion (hereby designated as (ATTTC)_58_) with the flanking AluJb element sequences that contain the right and the left monomers (RM and LM, respectively), have previously been cloned into T7-pCDH-CMV-MCS-EF1-GFP-T2A-Puro vectors and used in our published work (Seixas et al., 2017). The length of each repeat sequence was confirmed by Sanger sequencing. To *in-vitro* synthesize the (AUUUU)_7_, (AUUUU)_139_, and (AUUUC)_58_ RNAs, these T7-pCDH-CMV-MCS-EF1-GFP-T2A-Puro DNA vectors were linearized with NotI restriction enzyme (Anza, Invitrogen) and purified with DNA Clean & Concentrator kit (ZYMO Research, Irvine, CA, USA). *In-vitro* transcription was performed with T7 RNA polymerase (Thermo Fisher Scientific), and RNA was then purified with RNA Clean & Concentrator kit (ZYMO Research), aliquoted and stored at −80°C.

### Zebrafish microinjection

To generate embryos for microinjections, wild-type strain AB adult zebrafish were crossed, and collected embryos were microinjected at one- to two-cell stage with ∼5 nl (AUUUU)_7_ or (AUUUU)_139_, or (AUUUC)_58_ RNA at 100 ng/µl, as described by Seixas et al. (2017), or with purified H_2_O using a Narishige IM-300 microinjector (Narishige International, London, UK). As RNAs were diluted in H_2_O, embryos microinjected with this vehicle were used as a control for microinjection toxicity (vehicle condition). As human repetitive RNA can be toxic for zebrafish, animals microinjected with the (AUUUU)_7_ were used as an RNA control for the *DAB1* repeat region in the reference human genome. To control RNA integrity, we monitored the embryo lethality rate and morphological malformations in the (AUUUC)_58_ RNA condition at 100 ng/µl in each microinjection session, according to Seixas et al. (2017). A positive toxicity control with the (AUUUC)_58_ RNA at 150 ng/µl was also used in all microinjection sessions, and the replicate experiment was accepted only when this positive control was lethal/toxic to >60% of the microinjected embryos. To assess microinjection efficiency, each embryo was co-microinjected with 5 nl of each RNA repeat and *GFP* mRNA at a final concentration of 100 ng/µl. Each independent microinjection session was considered as an experimental replicate. Embryos were maintained at 28.5°C in Petri dishes containing embryo medium (E3) (5 mM NaCl, 0.17 mM KCl, 0.33 mM CaCl_2_, 0.33 mM MgSO_4_, 0.00015% Methylene Blue), supplemented with 0.003% 1-phenyl-2-thiourea (PTU) for imaging purposes, until 144 h post fertilization (hpf). Embryonic developmental stages were assessed based on morphological features corresponding to somite number, according to Kimmel et al. (1995). After 144 hpf, the microinjected animals were raised under a 14 h:10 h light:dark cycle, the first month in 1-l containers and then in 3.5-l tanks placed in a water-recirculating system under controlled conditions (temperature ∼27°C, pH ∼7, conductivity ∼900 µS). Adult animals were maintained at a maximum density of 8 fish/l per tank.

### RT-PCR amplification of microinjected RNA repeats

To determine the temporal profile of microinjected RNA repeats in zebrafish, total RNA from pooled embryos microinjected with vehicle, (AUUUU)_7_, (AUUUU)_139_, or (AUUUC)_58_ RNAs was extracted using the Quick-RNA MiniPrep Plus kit (ZYMO Research). After removing genomic DNA by DNase I treatment (Thermo Fisher Scientific), the RNAs were purified using the RNA Clean & Concentrator kit (ZYMO Research), and the RNA integrity was evaluated on the 2100 Bioanalyzer (Agilent). Then, 1 µg of each RNA was reverse transcribed (RT) by SuperScript III Reverse Transcriptase (Thermo Fisher Scientific), using random hexamers. Amplification of the repeat region specific to the microinjected human RNA was performed with 1 µl of cDNA, 200 µM of each dNTP, 1× PrimeSTAR GXL buffer Mg^2+^ Plus, 0.31 U PrimeSTAR GXL DNA polymerase (Takara Bio) and 4 µM of primers (STR24 GXL F1 and STR24 GXL R; Table S2) in a final volume of 20 µl. cDNAs underwent 1 min of initial denaturation at 98°C, followed by 30 cycles of amplification (98°C for 10 s and 68°C for 4 min) and 10 min of final extension at 68°C. RT-PCR-amplified products were detected by agarose gel electrophoresis.

### Zebrafish developmental arrest and malformations

To assess zebrafish developmental arrest and malformations, at least 100 embryos per experimental condition and replicate were microinjected, and unbiased analysis of embryo morphology was carried out at 24-26 hpf using a Leica KL 300 LED stereomicroscope (Leica Microsystems). Dead embryos were identified by lack of heartbeat and blood flow, and were counted and removed. Embryos were scored according to the severity of abnormalities in head and tail, with score 0 for no morphological defects, score 1 for tail defects, score 2 for head and tail defects, and score 3 for developmental arrest. To acquire representative images for morphological score analysis, embryos were anesthetized with 0.168 mg/ml tricaine (ethyl 3-aminobenzoate methanesulfonate) and images were taken at magnifications 2×, 5× or 8× using a stereomicroscope Leica M205 FA (Leica Microsystems) equipped with an Orca Flash 4.0LT imaging system (C11440-42U30, Hamamatsu Photonics). The number of embryos that survived and hatched was assessed daily from 0 to 96 hpf. At 24 hpf, the score-0 embryos with no morphological phenotype (undistinguishable from the wild type) were selected for further analyses.

### Whole-mount immunofluorescence

Whole-mount immunofluorescence was performed at 24 hpf, 72 hpf, and 120 hpf in zebrafish animals. For axon analysis, microinjected embryos at 24 hpf and larvae at 72 hpf were fixed for 3 h at 4°C in 4% PFA in PBS-T (0.1% Triton X-100 in PBS) and washed three times with PBS-T. For permeabilization and blocking, they were incubated in 1% Triton X-100 in PBS for 2 h at room temperature (RT) and then in 1% BSA 1% DMSO 1% NGS 0.7% Triton X-100 in PBS for 1 h at RT. Then, samples were incubated with anti-synaptic vesicle 2 (SV2) antibody [1:200, mouse, AB2315387, Developmental Studies Hybridoma Bank (DSHB)], diluted in blocking solution overnight at 4°C. Then, animals were washed three times with PBS-T and incubated with the secondary antibody Alexa Fluor-647 goat anti-mouse IgG (1:750, A-21235, Thermo Fischer Scientific) and DAPI (1:1000, Sigma-Aldrich, Merck), diluted in blocking solution for 4 h at RT. For the analysis of neuromuscular junctions (NMJs), embryos and larvae were also incubated with the postsynaptic acetylcholine receptor (AChR) marker tetramethylrhodamine-conjugated α-bungarotoxin (α-BTX, 1:1000, T1175, Thermo Fischer Scientific) diluted in blocking solution for 4 h at RT. Embryos were washed three times with PBS-T and mounted on microscope slides using glycerol-based ibidi mounting medium (50011, ibidi). For zebrin II-positive Purkinje cell analysis, larvae were fixed at 120 hpf and incubated with an anti-zebrin II antibody (1:50, kindly gifted by Richard B. Hawkes, Department of Cell Biology & Anatomy, and Hotchkiss Brain Institute, Cumming School of Medicine, University of Calgary, Alberta, Canada.) (Lannoo et al., 1991a,b) and the secondary antibody Alexa Fluor-647 goat anti-mouse IgG (1:750, A-21235, Thermo Fischer Scientific), under the conditions described above. Images were acquired on an inverted Leica SP8 single-point scanning confocal microscope equipped with a fully motorized DMi8 microscope (Leica Microsystems) a HC PL APO 40×/1.10 Water CS2 (with motorized correction ring) objective lens (see Supplementary Materials and Methods). For zebrin II-positive Purkinje cell analysis, the same system was used with a 25× objective (NA 0.95; see Supplementary Materials and Methods). To analyze axonal length of primary motor neurons (PMNs), the six axons in the six-somite-spanning region immediately anterior to the cloaca were measured to guarantee analysis of the same axons in all zebrafish groups; an average-intensity z-projection of at least five microinjected zebrafish embryos per experimental condition and replicate was used to perform the axon tracing by using Fiji plugin NeuronJ (https://imagej.net/plugins/neuronj) (Meijering et al., 2004; Schindelin et al., 2012). Briefly, axon tracing was performed manually from the closest point of the neuron soma to the end of the axonal terminal, and average axonal length was calculated for each embryo. For colocalization analysis of presynaptic PMN axons (labeled with anti-SV2 antibody) and postsynaptic AChRs (marked with α-BTX), images of at least five previously microinjected embryos and larvae from each condition and replicate were deconvolved by using the Deconvolution Express module and the Standard algorithm from Huygens Professional (https://svi.nl/Deconvolution-Express), followed by the calculation of Pearson's colocalization coefficient (version 20.04, SVI). The distribution of AChRs in the dorsal-ventral axis of muscle in zebrafish embryos and larvae was assessed in z-projections (maximum intensity) using the Fiji plugin NeuronJ (Meijering et al., 2004; Schindelin et al., 2012), starting the measurement in the horizontal myoseptum for both dorsal and ventral orientations. The number of AChRs was determined by staining for α-BTX using the Object Analyzer tool from Huygens Professional and the SV2 staining as a reference for segmentation (version 20.04, SVI). To determine the area occupied by AChRs within NMJs, a z-maximum intensity projection was made to manually define the regions of interest based on TRITC-conjugated α-BTX-staining of AChRs. For analysis of Purkinje cells, IMARIS software (version 10.2.0, Andor Oxford) was used to determine the volume and mean intensity of zebrin II-positive cells in at least nine larvae from each condition per replicate.

### Spontaneous tail coiling assay

Involuntary tail coiling begins ∼17 hpf and embryos become touch-responsive by 27 hpf (Drapeau et al., 2002); therefore, spontaneous tail coiling of zebrafish was assessed at 24 hpf. Microinjected embryos with ∼30-somites morphology were placed in Petri dishes and acclimatized to room light for 30 min. The number of tail movements per embryo for 60 s was counted by observers unaware of embryo conditions, under a Leica KL 300 LED stereomicroscope. Representative videos of spontaneous coiling were acquired using a stereomicroscope Leica M205 FA (Leica Microsystems) equipped with an Orca Flash 4.0 LT imaging system (model C11440-42U30, Hamamatsu Photonics) controlled using the Micro-Manager Fiji plugin (Edelstein et al., 2010).

### Touch-evoked escape swimming response test

To test the escape response to a tactile stimulus, at least 20 larvae at 72 hpf were tested by observers unaware of the embryo condition (with a total of six replicates), when larvae already exhibit sustained swimming behavior in response to touch (Drapeau et al., 2002). For acclimatization, microinjected embryos at 24 hpf, were placed in a room with controlled temperature (∼27°C) and a 14 h:10 h light:dark cycle. The animals had been acclimatized to the intensity of the light box for at least 30 min before the test was started at 72 hpf. Then, each larva was placed in a Petri dish on top of a light box with a white acrylic panel to reduce light intensity. Once the free-swimming movement stopped, each larva was gently touched on the tail with a black filament. Swimming responses were recorded using a GoPro Hero8 Black camera (serial no.: C3331350868456, GoPro) at 120 frames per second until the larva stopped swimming. Touch-evoked escape response was assessed as no response or presence of response (any movement or swimming response). Also, the reaction time between touch and start of the response, and the swimming duration (time that larvae swim after touch) were analyzed. The swimming response is characterized by a movement away from the touch-escape response. The reaction time and swimming duration were analyzed frame by frame using Fiji software (Schindelin et al., 2012). Larvae that did not show any response were excluded from analyses of reaction time and swimming duration; larvae that swam to the border of the Petri dish were excluded from analysis of the swimming duration.

### Spontaneous locomotor behavior test

To assess spontaneous motor activity, the spontaneous locomotor activity test was performed in 20 larvae per condition and replicate at 144 hpf in six-well plates (three replicates). At this stage, larvae already show autonomous feeding and sustained free-swimming behavior (Drapeau et al., 2002). For each well, 5 ml of 0.5% agarose diluted in the fish-recirculating water system was polymerized and a circular hole (radius ∼1.9 mm) was made in the center of each agarose well. The wells were filled with 2 ml of water from the fish-recirculating system. Larvae from each experimental condition were randomized and added into wells, and analysed by an observer unaware of the experimental conditions. Locomotor activities of larvae were recorded with a GoPro Hero8 Black (serial no.: C3331350868456, GoPro) at 60 frames per second for 10 min, at 2× magnification. Larvae tracking was performed using ANY-maze software (https://www.any-maze.com/) (Stoelting Europe, Dublin, Ireland). Locomotor activity was assessed by measuring the immobility per 10 min (in %), swimming distance (in cm), average speed (in cm/s), time immobile (in s) and number of immobile episodes. Immobility was considered when no movement was detected for >2 s. If correct tracking was not possible, movies were discarded.

### Novel diving tank test

The novel diving tank test was performed with 3- and 17-month-old animals (young and late adulthood, respectively) to assess locomotor activity in adult fish. Microinjected embryos without morphologic malformations from five independent microinjection sessions were raised. Approximately 25 adult fish per condition were placed in 3.5-l tanks. For the novel diving tank test, each fish was placed in a 3.5-l tank filled with 2.5 l of fresh water (12 cm of water column) from the fish-recirculating system, and locomotor activity was recorded at 25 frames per second for 6 min using a side-view camera (Canon Legria HF R606). Movies were converted from MP4 to AVI format using Any Video Converter software and fish tracking was performed using the open-source software TheRealFishTracker (https://www.dgp.toronto.edu/~mccrae/projects/FishTracker/) (Buske and Gerlai, 2014). Immobility (in %), swimming distance (in m) and average speed (in cm/s) were calculated for each fish by using the coordinates provided by the software. After the test, animals were euthanized with 0.300 g/l of tricaine, and standard length and weight measured per fish. Brains were collected for further histological analysis.

### Zebrafish brain histology and immunofluorescence

Zebrafish brains were dissected and fixed overnight in 4% PFA in PBS at 4°C, followed by three washes with PBT (PBS with 0.1% Tween) and stored in methanol at −20°C. Tissues were pre-processed by dehydration in ethanol at increasing concentrations, clearance with xylene and a final step with paraffin, for 10 min in each reagent. Brains were embedded in paraffin, and 5-µm slices were longitudinally sectioned using a Leica RM2255 microtome (Leica Biosystems). Before staining and immunofluorescence, deparaffinization and rehydration of slices were performed in reverse order compared with the previous protocol. Then, slices were stained with hematoxylin and eosin or underwent an anti-zebrin II antibody immunofluorescence protocol. For immunofluorescence, slices were submitted to antigen retrieval with 10 mM EDTA pH 8.0 in a steamer for 40 min, followed by 20 min at RT. Immunofluorescence with anti-zebrin II antibody (1:50; kindly gifted by Dr Richard B. Hawkes) (Lannoo et al., 1991a,b) was performed as described above. Imaging was performed using the laser spinning disk confocal microscope Andor BC43 (Andor Oxford Instruments), equipped with a CFI Plan Apochromat Lambda D 20X/0.8 DIC (see Supplementary Materials and Methods); a minimum of three animals were analyzed per condition. Hematoxylin and eosin-stained slices were digitized using the Phenoimager™ HT (Akoya Biosciences, Marlborough, MA, USA), and the area of the cerebellar molecular cell layer was measured in ten consecutive slices for each brain by using the QuPath-0.5.1 (Bankhead et al., 2017). The average number of zebrin II-positive Purkinje cells was quantified in five consecutive slices of each brain by using anti-zebrin II fluorescence in IMARIS software (version 10.2.0, Andor Oxford Instruments).

### RBP *in silico* analysis

To identify potential RBPs that bind to the non-pathogenic and SCA37-associated (AUUUC)_58_ RNAs, the RBPmap webserver (https://rbpmap.technion.ac.il/) was used (Paz et al., 2014). Given the complete length of the pathogenic repeat allele of ∼199 repeat units (Seixas et al., 2017), the non-pathogenic (AUUUU)_199_ sequence was introduced into this database as well as the complete repeat sequence of the pathogenic (AUUUU)_57_(AUUUC)_58_(AUUUU)_84_, which corresponds to its abbreviated name (AUUUC)_58_ RNA. All human/mouse RBP motifs from the database were selected, and default stringency and conservation filter applied. From the z-scores of each predicted binding site (*P*<0.05), the maximum z-score was determined for each RBP hit. The number of putative binding sites for each RBP was calculated for non-pathogenic and SCA37-associated (AUUUC)_58_ RNAs. This analysis was performed to identify differences in the binding affinity of each RBP between non-pathogenic and SCA37-associated (AUUUC)_58_ RNAs.

### Human NOVA2 experiments in zebrafish

To synthesize human *NOVA2* mRNA for microinjection in zebrafish, a sequence encoding fourteen histidine residues was cloned in pCMV6-XL6-NOVA2 (SC303210, Origene, Rockville, MD, USA) at the C-terminal of NOVA2 cDNA by using the IVA cloning approach (García-Nafría et al., 2016), for NOVA2 immunofluorescence staining with anti-histidine antibody. Amplification of *NOVA2* was performed using KOD DNA polymerase (Sigma-Aldrich) with the forward His-tag_Fw and reverse His-tag_Rv primers (Table S2). Human *NOVA2* mRNA was *in vitro* synthesized from the pCMV6-XL6-NOVA2-HisTag DNA vector. After DNA plasmid linearization with XbaI restriction enzyme (Anza, Invitrogen) and purification with DNA Clean & Concentrator kit (ZYMO Research, Irvine, CA, USA), *NOVA2* cDNA was *in vitro* transcribed with SP6 RNA polymerase (Thermo Fisher Scientific), and the mRNA was purified with RNA Clean & Concentrator kit (ZYMO Research), aliquoted and stored at −80°C. Zebrafish embryos were microinjected and maintained as described above. Translation of the human NOVA2 protein in zebrafish was confirmed by whole-mount immunofluorescence with anti-histidine-tagged antibody (1:500, 05-949, Millipore) in 24 hpf zebrafish embryos. Briefly, embryos were fixed for 3 h at 4°C in 4% PFA in PBS-T, washed three times with PBS-T, and permeabilized with 1% Triton X-100 in PBS for 2 h at RT. Samples were blocked with 5% BSA in PBS-T for 1 h at RT and incubated with anti-histidine-tagged antibody (1:500, 05-949, Millipore) diluted in blocking solution overnight at 4°C. Then, embryos were washed three times with PBS-T and incubated with the secondary antibody Alexa Fluor-647 goat anti-mouse IgG (1:750, A-21235, Thermo Fischer Scientific) and DAPI (1:1000, Sigma-Aldrich, Merck) diluted in blocking solution for 4 h at RT. Whole-mount immunofluorescence staining with anti-SV2 antibody and analysis were also performed in 24 hpf embryos, as described above. Images were acquired on an inverted Leica SP8 single-point scanning confocal microscope equipped with a fully motorized DMi8 microscope (Leica Microsystems) a HC PL APO 40×/1.10 Water CS2 (with motorized correction ring) objective lens (see Supplementary Materials and Methods).

### Zebrafish *nova2* morpholino-mediated knockdown

Embryos from wild-type strain AB were microinjected with ∼6.7 ng of *nova2* or standard control morpholino oligomer (Table S3) as described above and according to Giampietro and colleagues (2015). This *nova2*-targeting morpholino oligonucleotide binds to the start codon and blocks Nova2 translation in zebrafish (Giampietro et al., 2015). Co-microinjection of 6.7 ng *nova2* morpholino and human *NOVA2* mRNA at 100 ng/µl was performed as described above. Whole-mount immunofluorescence staining with anti-SV2 antibody and analysis were performed in microinjected embryos at 24 hpf, as described above.

### Validation of Nova2 knockdown in zebrafish

Total RNA was extracted from pooled microinjected embryos and reverse transcription (RT) was carried out as described above; and the extracted RNA integrity was evaluated on the 2100 Bioanalyzer (Agilent). The RT-PCR amplification of each target was performed with 1 µl of cDNA, 1.25 U NZYTaq II 2× Green Master Mix (NZYTech, Lisbon, Portugal), and 4 µM of primers as described (Giampietro et al., 2015; Jelen et al., 2007) (see Table S2) in a final volume of 20 µl. cDNAs were subjected to an initial denaturation at 95°C for 5 min, followed by 30 cycles of amplification (95°C for 30 s, 60°C for 30 s, 72°C for 40 s) and a final extension at 72°C for 10 min. The PCR products were analyzed on a 2100 Bioanalyzer (Agilent). At least three biological replicates from independent microinjection sessions were analyzed for each condition. Alternative splicing isoforms were confirmed by Sanger sequencing.

### Statistical analysis

GraphPad Prism was used for the graphical representation of data, and statistical analysis was performed using IBM SPSS. Shapiro-Wilk and Levene's tests were used to assess the normality of data and homogeneity of variances, respectively. Data were transformed (logarithmic or cube root transformation) when not normally distributed. If transformed data were not normal, non-parametric tests were used. The χ^2^ test was used for *n*≥50 to compare categorical variables. To evaluate differences among experimental groups, one-way analysis of variance (ANOVA) following Bonferroni post-hoc test was used for normally distributed data, and Kruskal–Wallis test following Dunn's test as a post-hoc test were used for non-normal data. When only the homogeneity of variances of the data was not verified, Welch's correction was applied to one-way ANOVA, and Dunnett's T3 post-hoc test was used. Two-way ANOVA was used to study how two independent variables (e.g. condition and age, condition and replicates or condition and sex) affect a dependent variable. For comparisons between two independent samples, independent Student's *t*-test or Mann–Whitney *U*-Test was used for normally or non-normally distributed data, respectively. The log-rank test was used for zebrafish survival and hatching rates. Percentage or mean values and standard deviation details are described in the results section. Sample size and number of replicates for each analysis are indicated in figure legends. Significant differences were considered when *P*≤0.05 (two-tailed test).

## Supplementary Material

10.1242/dmm.052636_sup1 Supplementary information
