# Supplementary material for: Embryonic spinocerebellar ataxia type 37-associated AUUUC repeat RNA causes neurodevelopmental defects
Source: Dis Model Mech. 2026 Apr 30;19(4):dmm052636. doi: 10.1242/dmm.052636 (PMC13225218; doi:10.1242/dmm.052636)
Supplement: Supplementary information [file dmm-19-052636-s1.pdf]

Supplementary Materials and Methods

Embryo/larva imaging on inverted Leica SP8 single point scanning confocal microscope

Zebrafish PMNs images were acquired with a 405 Diode laser line, a tunable Argon laser AOBS modulated and 633 HeNe laser line, and Leica PMT and HyD detectors. PMNs were imaged sequentially by line scanning unidirectionally at 400 Hz using the galvanometer-based imaging mode and line average of 3, with a pixel size of 0.28 µm and a z step-size of 0.42 µm, for an area size of 290.62 µm x 290.62 µm in Leica LasX software (version 3.5.6.21594) and saved as LIF files. For all images the pinhole size was 77.2 µm, calculated at 1 AU for 580 nm emission. DAPI was excited with 405 nm laser line with laser power of 2.99%, and emission was collected on a Leica PMT2 detector with a collection window of 415 – 478 nm with 601.6 V and an offset of 0. AlexaFluor 647 was excited with 633 nm laser line with laser power of 0.10%, and emission was collected on a Leica HyD4 (standard mode) detector with a collection window of 643 – 743 nm and smart gain of 19.4%. Zebrafish NMJs were imaged sequentially by line scanning bidirectionally at 400 Hz using the galvanometer-based imaging mode and line average of 5, with a pixel size of 0.19 µm and a z step-size of 0.42 µm, for an area size of 193.75 µm x 193.75 µm in Leica LasX software (version 3.5.6.21594) and saved as LIF files. DAPI and AlexaFluor 647 were excited as described for PMNs. Tetramethylrhodamine was excited with 514 nm laser line with laser power of 0.50% (Argon at 29.31%), and emission was collected on a Leica HyD3 detector with a collection window of 524 – 625 nm with a smart gain of 51.6%. Purkinje cell images of 120 hpf larvae were acquired sequentially by line scanning unidirectionally at 400 Hz using the galvanometer-based imaging mode and line average of 3, with a pixel size of 0.38 µm and a z step-size of 0.57 µm, for an area size of 387.5 µm x 387.5 µm in Leica LasX software (version 3.5.6.21594) and saved as LIF files. For all images the pinhole size was 55.7 µm, calculated at 1 AU for 580 nm emission. DAPI was excited with 405 nm laser line with laser power of 1.50%, and emission was collected on a Leica PMT2 detector with a collection window of 415– 478 nm with 600 V and an offset of 0. AlexaFluor 647 was excited with 633 nm laser line with laser power of 0.10%, and emission was collected on a Leica HyD3 detector with a collection window of 643 – 696 nm and smart gain of 50%. To assess human NOVA2 protein translation in zebrafish, images were acquired sequentially by line scanning bidirectionally at 400 Hz using the galvanometer-

Disease Models & Mechanisms • Supplementary information

based imaging mode and line average of 3, with a pixel size of 0.03 µm and a z step-size of 0.42 µm, for an area size of 29.06 µm x 29.06 µm in Leica LasX software (version 3.5.6.21594) and saved as LIF files. For all images the pinhole size was 77.2 µm, calculated at 1 AU for 580 nm emission. DAPI was excited with 405 nm laser line with laser power of 2.99%, and emission was collected on a Leica PMT2 detector with a collection window of 415 – 478 nm with 601.6 V and an offset of 0. AlexaFluor 647 was excited with 633 nm laser line with laser power of 0.10%, and emission was collected on a Leica HyD4 (standard mode) detector with a collection window of 643 – 743 nm and smart gain of 19.4%. In NOVA2 rescue experiments, images were acquired with a 405 Diode laser line and 633 HeNe laser line, and Leica PMT and HyD detectors. PMNs were imaged sequentially by line scanning bidirectionally at 400 Hz using the galvanometer-based imaging mode and line average of 3, with a pixel size of 0.28 µm and a z step-size of 0.42 µm, for an area size of 290.62 µm x 290.62 µm in Leica LasX software (version 3.5.6.21594) and saved as LIF files. For all images the pinhole size was 77.2 µm, calculated at 1 AU for 580 nm emission. DAPI was excited with 405 nm laser line with laser power of 4.09%, and emission was collected on a Leica PMT2 detector with a collection window of 415 – 478 nm with 601.6 V and an offset of 0. AlexaFluor 647 was excited with 633 nm laser line with laser power of 0.30%, and emission was collected on a Leica HyD4 (standard mode) detector with a collection window of 643 – 743 nm and smart gain of 19.4%.

Adult brain slices imaging on laser spinning disk confocal microscope Andor BC43

DAPI was excited with 405 nm laser line with laser power of 100%, with an exposure time of 50 ms, and emission was collected with a 445/20 filter. AlexaFluor 647 was excited with 638 nm laser line with laser power of 90%, with an exposure time of 50 ms, and emission was collected by a 708/75 filter.

Disease Models & Mechanisms • Supplementary information

A

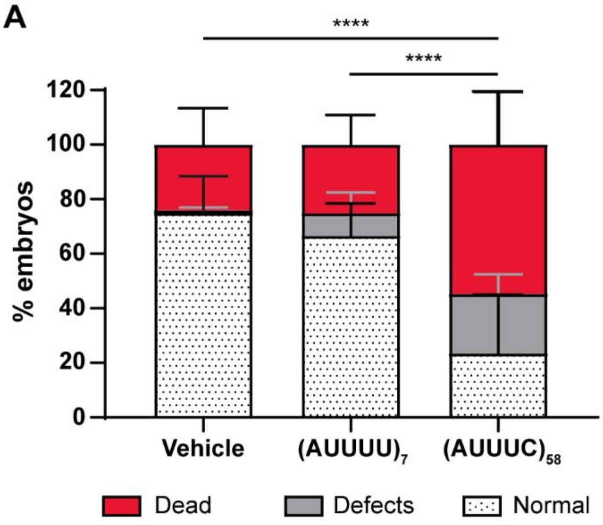

C

Condition \ Score	0	1	2	3
Vehicle	98.81	0.38	0.67	0.14
$(AUUUU)_7$	89.53	7.90	2.39	0.18
$(AUUUC)_{58}$	47.22	24.63	26.99	1.16

B

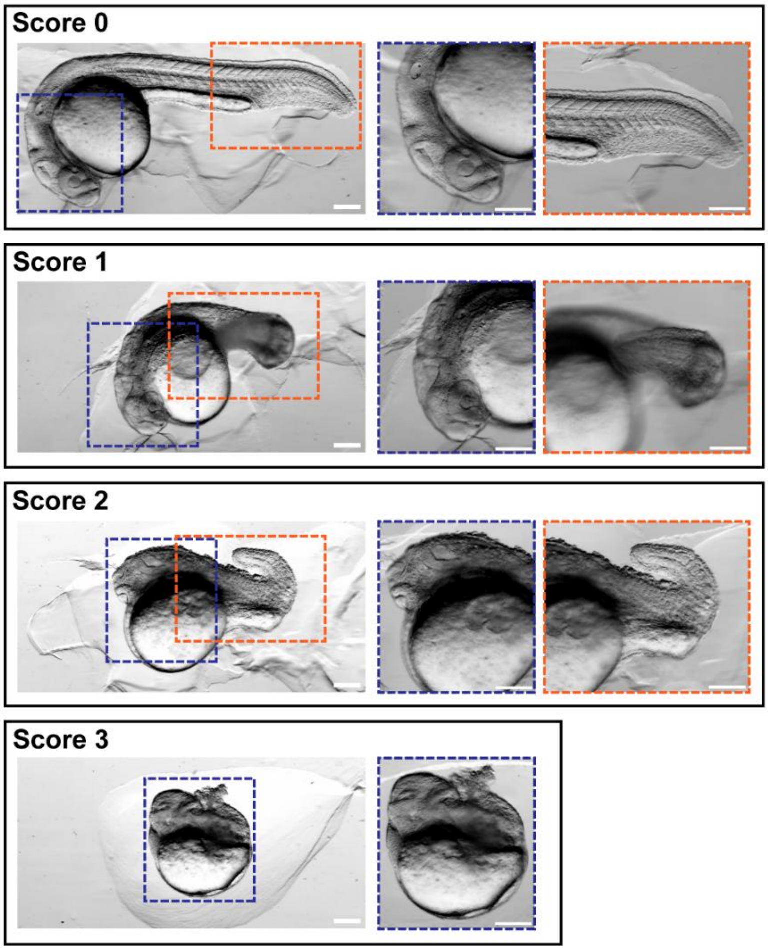

Fig. S1 The $(AUUUC)_n$ insertion RNA causes developmental toxicity, resulting in arrested development and lethality. Related to **Fig. 1**. (A) Percentage of dead (red), defective (gray) and normal (white with black dots) embryos observed at 24 hpf following the microinjection of vehicle,

Disease Models & Mechanisms • Supplementary information

$(AUUUU)_7$ or $(AUUUC)_{58}$ RNAs (average of eight independent experiments with at least 100 embryos per experiment; ****$p<0.0001$, χ^2 test for lethality and morphological defects). Data are shown as mean ± standard deviation. (B) Representative images correspond to the phenotypical scores, 0 - embryos without morphological defects, 1 - embryos with defects in tail, 2 - embryos with defects in tail and head, and 3 - arrested embryonic development; respective zoom-in images are presented for blue and orange delimitations; scale bar = 50 μm. (C) Percentage of embryos exhibiting different phenotypical scores.

Disease Models & Mechanisms • Supplementary information

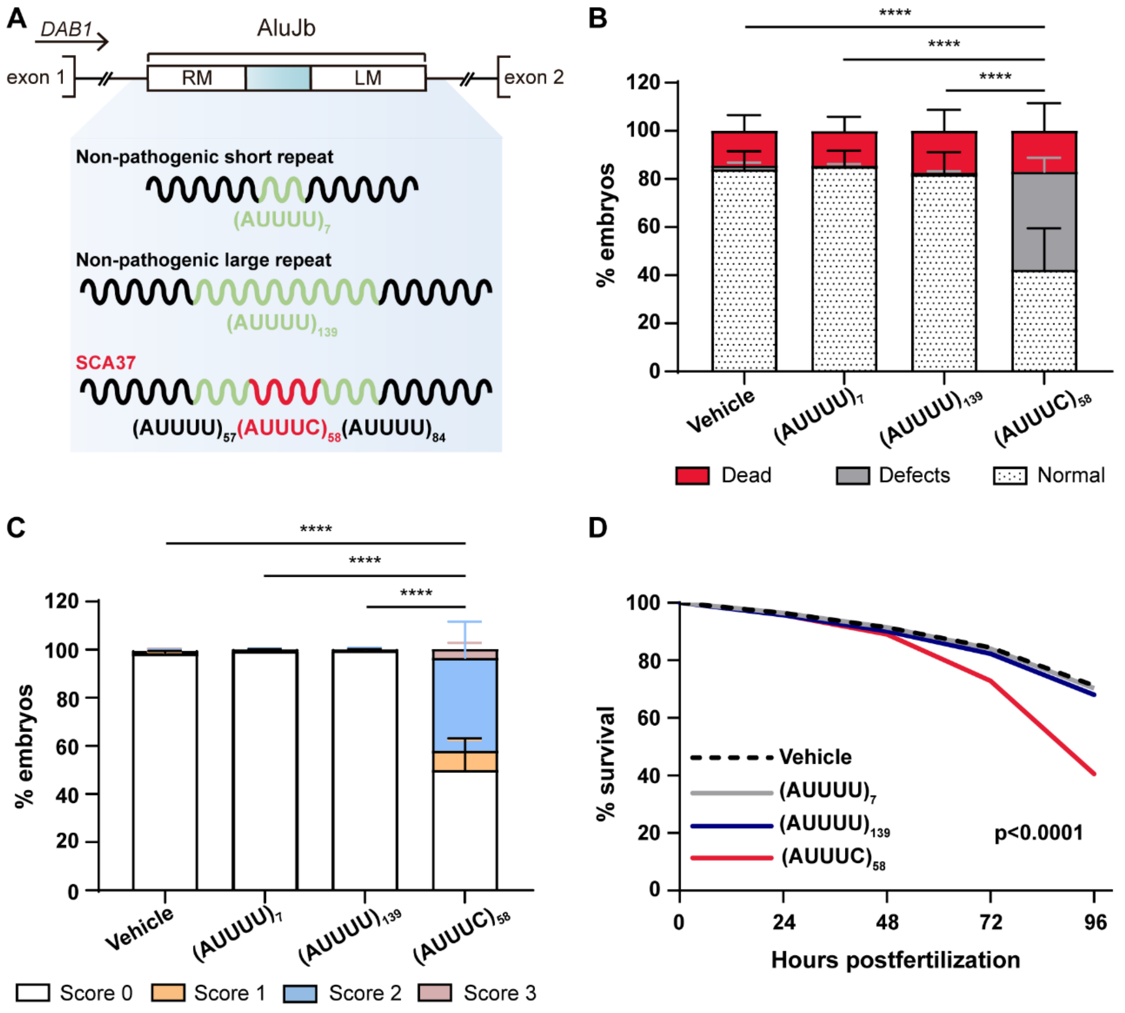

Fig. S2 The (AUUUC)$_n$ RNA motif, rather than large AUUUU repeat RNAs, triggers the observed embryonic RNA toxicity and morphological defects. Related to **Fig. 1**. Data from three independent experiments after microinjection of 100 embryos per condition, including the vehicle, (AUUUU)$_7$, (AUUUU)$_{139}$, or (AUUUC)$_{58}$ RNAs. (A) A schematic representation depicts the microinjected RNAs, including the non-pathogenic short and large repeats with 7 and 139 AUUUU repeat units, respectively, and the pathogenic AUUUC repeat insertion, flanked by AluJb monomers. (B) Percentage of dead (red), defective (gray) and normal (white with black dots) embryos at 24 hpf after microinjection of vehicle, (AUUUU)$_7$, (AUUUU)$_{139}$, or (AUUUC)$_{58}$ RNAs (****$p<0.0001$, χ^2 test for lethality and morphological defects). Data are shown as mean ± standard deviation. (C) Percentage of embryos scored from 0 to 3 according to the severity of morphological phenotype at 24 hpf (score 0, no morphological defects; score 1, tail defects; score 2, head and tail defects; and score 3, developmental arrest). Statistical analysis showed

Disease Models & Mechanisms • Supplementary information

significant differences across conditions (****$p<0.0001$, Kruskal-Wallis test followed by Dunn's post-hoc test for scored embryos). Data are presented as mean ± standard deviation. (D) Survival rate from 0 to 96 hpf was monitored after microinjection of vehicle, $(AUUUU)_7$, $(AUUUU)_{139}$, or $(AUUUC)_{58}$ RNAs. Statistically significant differences were observed between the vehicle or control RNA repeats and the pathogenic $(AUUUC)_{58}$ RNA (****$p<0.0001$, Log Rank test for survival).

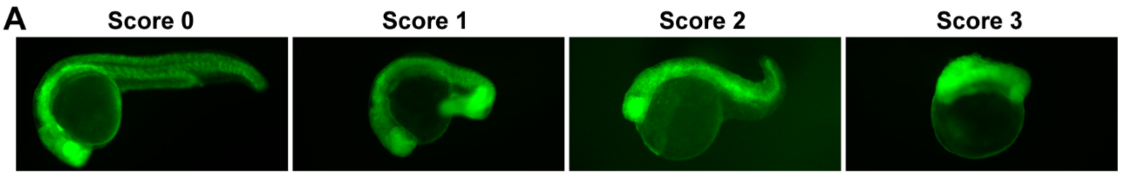

B

Condition \ Phenotype	Normal embryos		Defected embryos	
Vehicle	98.67	1.33	100	0
$(AUUUU)_7$	99.33	0.67	100	0
$(AUUUU)_{139}$	99.67	0.33	100	0
$(AUUUC)_{58}$	98.83	1.17	100	0

Percentage of embryos: GFP positive (green); GFP negative (red)

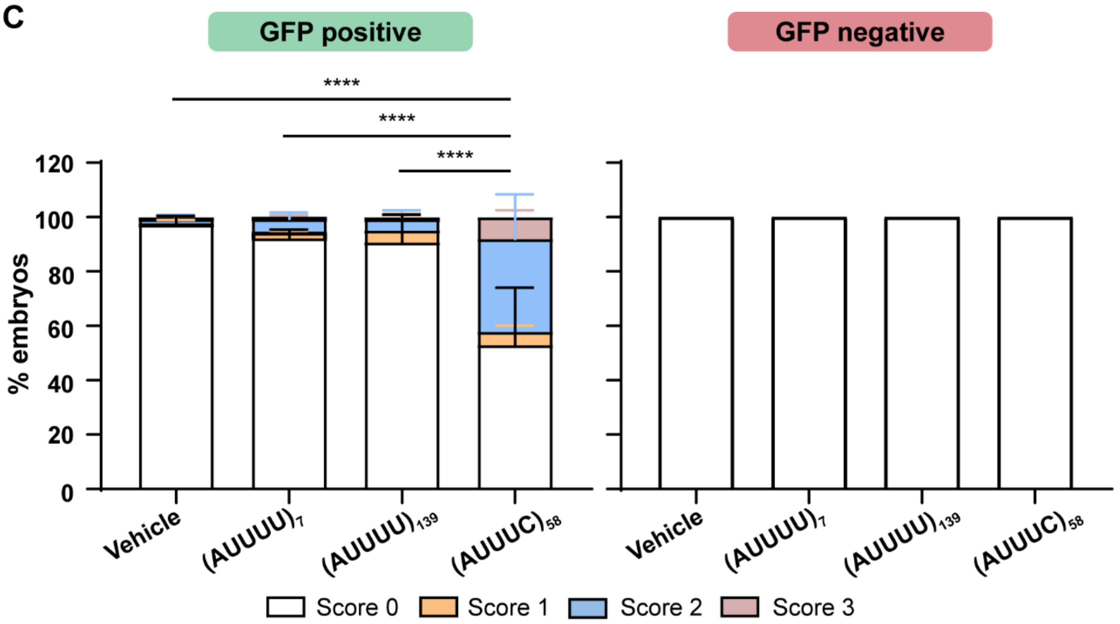

Fig. S3. The co-microinjection of RNA repeats and *GFP* mRNA was successful in the vast majority of zebrafish embryos at 24 hpf. Related to **Fig. 1**. Data from three independent experiments with 200 embryos co-microinjected with *GFP* mRNA and vehicle, $(AUUUU)_7$, $(AUUUU)_{139}$, or $(AUUUC)_{58}$ RNAs. (A) Representative images showing a broad GFP expression throughout the embryo across all phenotypical scores (score 0, no morphological defects; score 1, tail defects; score 2, head and tail defects; and score 3, developmental arrest). (B) The percentage of embryos expressing GFP in both normal and defective embryos indicates high microinjection efficiency. (C) Percentage of embryos expressing (left) or lacking (right) GFP in each phenotypical score. Statistical analysis revealed significant differences between control conditions and the $(AUUUC)_{58}$ RNA (****$p<0.0001$, Kruskal-Wallis test followed by Dunn's post-hoc test for scored embryos). Data are shown as mean ± standard deviation.

Disease Models & Mechanisms • Supplementary information

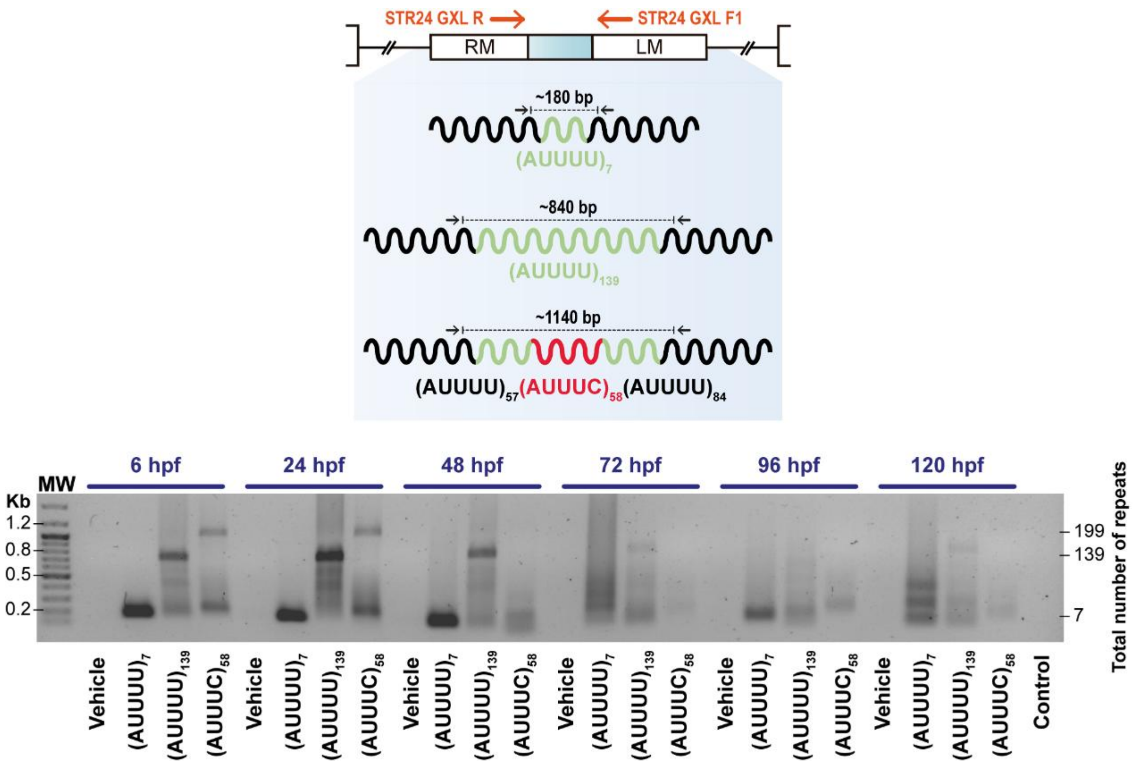

Fig. S4. Temporal profile of the microinjected RNA repeats in zebrafish. Related to **Fig. 1F**. RNA was extracted from pooled embryos after microinjection of vehicle, $(AUUUU)_7$, $(AUUUU)_{139}$, or $(AUUUC)_{58}$ RNA, at 6, 24, 48, 72, 96, and 120 hpf. (top) Schematic representation of the repeat region and the flanking primers used for RT-PCR (orange arrows) with the respective expected product lengths. (bottom) Full gel electrophoresis, with overlap with Fig. 1F for 6 and 24 hpf, shows amplification of the repeat region, demonstrating that the microinjected RNAs remain detectable in zebrafish up to 120 hpf, although they exhibit pronounced degradation at these later stages.

Disease Models & Mechanisms • Supplementary information

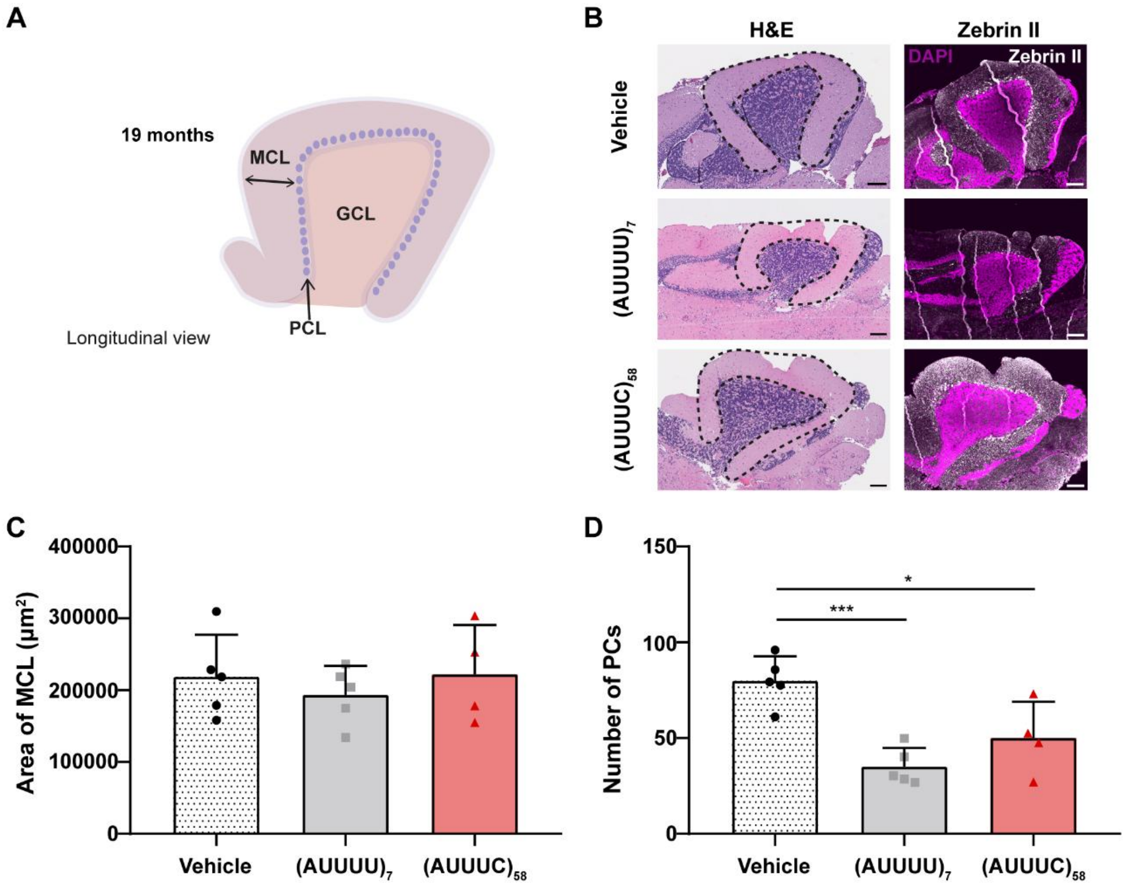

Fig. S5 No evidence of zebrin II-positive Purkinje cell loss in adult animals previously microinjected with the $(AUUUC)_{58}$ RNA. (A) The schematic representation illustrates the adult zebrafish cerebellum from a longitudinal view, highlighting the anatomical layout and the location of Purkinje cells within the cerebellar structure. This representation serves as a foundational overview for comparing Purkinje cell presence and distribution in RNA microinjected versus control animals. (B) Representative images depict paraffin-embedding brain sections of 19-month-old zebrafish. The left panel shows hematoxylin and eosin staining, providing a general view of the cellular architecture within the cerebellum; all the cerebellar molecular cell layer delimited in a black dashed line was measured, independently of brain size. The right panel displays immunofluorescence results using an anti-zebrin II antibody, counterstained with DAPI to highlight cell nuclei. Scale bar = 100 µm. (C) The area of the cerebellar molecular cell layer was assessed with sample sizes consisting of n=5 animals in the vehicle group, n=5 animals in the $(AUUUU)_7$ group, and n=4 animals in the $(AUUUC)_{58}$ group. Statistical analysis via One-way ANOVA indicated no significant differences in the area among the conditions. (D) The number of

Disease Models & Mechanisms • Supplementary information

Purkinje cells was quantified across the different animal groups, with n=5 animals in the vehicle group, n=5 animals in the $(AUUUU)_7$ group, and n=4 animals in the $(AUUUC)_{58}$ group. The analysis revealed a significant difference in Purkinje cell number, indicated by a p-value of *$p<0.05$ and ***$p<0.001$, as determined by One-way ANOVA followed by Bonferroni correction. Data are shown as mean ± standard deviation. Abbreviations: PC – Purkinje cells; GCL – granular cell layer; PCL – Purkinje cell layer; MCL – molecular cell layer.

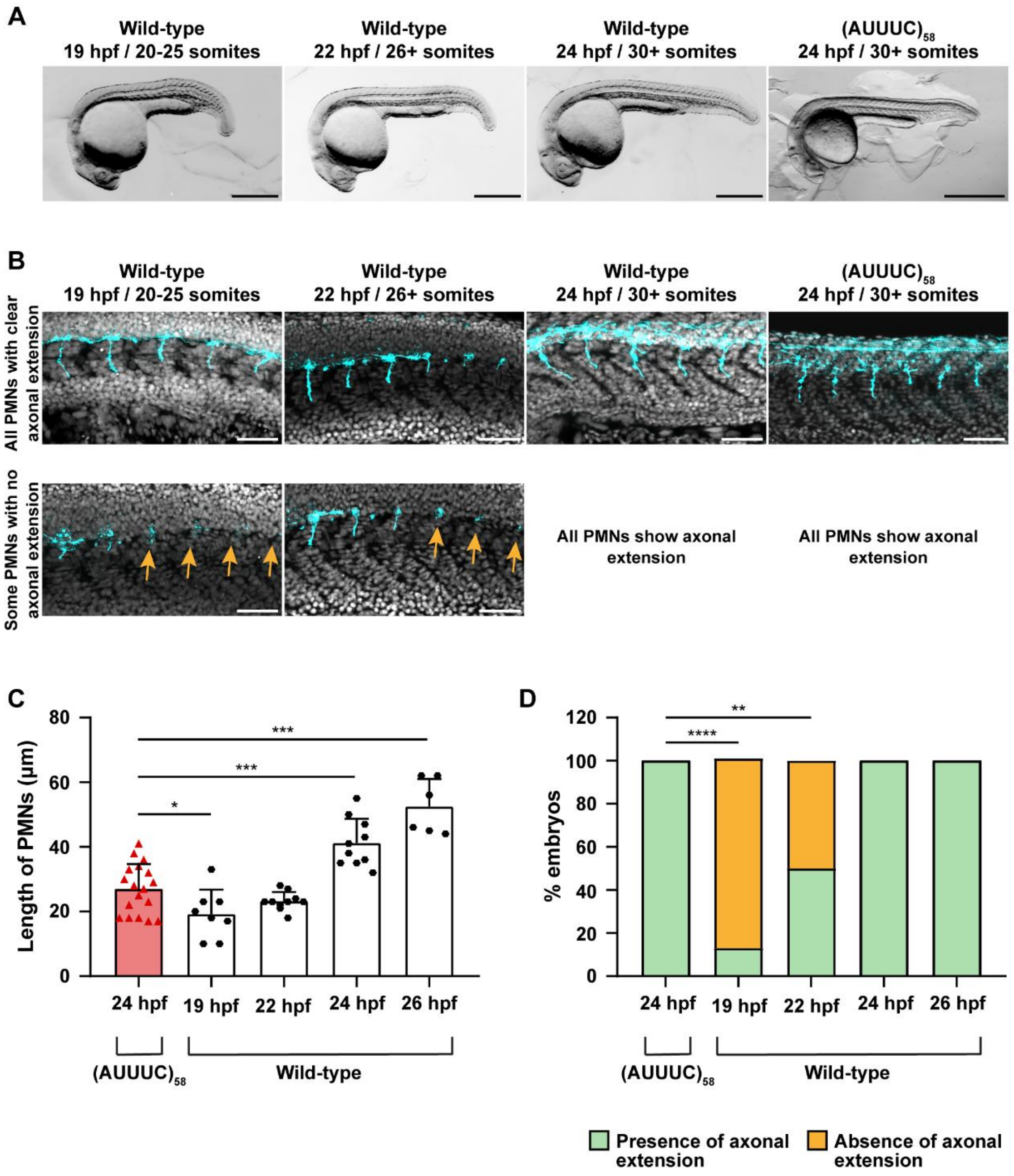

Fig. S6 The (AUUUC)$_{58}$-injected embryos show axonal extension of PMNs at 24 hpf. Related to **Fig. 3B.** (A) Representative images showing the morphology of non-injected wild-type embryos at 19, 22 and 24 hpf and (AUUUC)$_{58}$-injected embryos at 24 hpf; microinjected embryos at 24 hpf used for PMN axonal length quantification; scale bar = 200 µm. (B) Representative z-average projection images of PMNs axons from non-injected wild-type embryos at 19, 22 and 24 hpf and from (AUUUC)$_{58}$-injected embryos at 24 hpf, in the 6-somites region anterior to the cloaca (except for 19 hpf, as only one out of eight embryos showed extension of PMNs; scale bar = 50 µm), demonstrating that some PMNs do not present axonal outgrowth at 19 and 22 hpf (orange

Disease Models & Mechanisms • Supplementary information

arrows), in contrast to 24 hpf. (C) Quantification of PMN axonal length in both $(AUUUC)_{58}$-injected and non-injected embryos at different timepoints. Statistical analyses showed significant differences between axonal length of PMNs in $(AUUUC)_{58}$ RNA microinjected embryos at 24 hpf and non-injected embryos at 19, 24 or 26 hpf (n=18 embryos microinjected with the $(AUUUC)_{58}$ RNA, n=8 wild-type embryos at 19 hpf, n=10 wild-type embryos at 22 hpf, n=10 wild-type embryos at 24 hpf, n=6 wild-type embryos at 26 hpf; *$p<0.05$ and ***$p<0.001$, independent Student's t-test). Data are represented as mean ± standard deviation. (D) Percentage of embryos presenting axonal extension of PMNs in the 6-somites region anterior to the cloaca at different timepoints. All the embryos microinjected with the $(AUUUC)_{58}$ RNA at 24 hpf show axonal extension, when compared with the non-injected wild-type embryos at 19 and 22 hpf. Statistical analysis demonstrated significant differences in terms of presence or absence of axonal extension between $(AUUUC)_{58}$-injected embryos and wild-type embryos at 19 or 22 hpf (n=18 embryos microinjected with the $(AUUUC)_{58}$ RNA, n=8 wild-type embryos at 19 hpf, n=10 wild-type embryos at 22 hpf, n=10 wild-type embryos at 24 hpf, n=6 wild-type embryos at 26 hpf; **$p<0.01$ and ****$p<0.0001$, Fisher's exact test for number of embryos with or without PMN axonal extension). Data represented as mean.

Disease Models & Mechanisms • Supplementary information

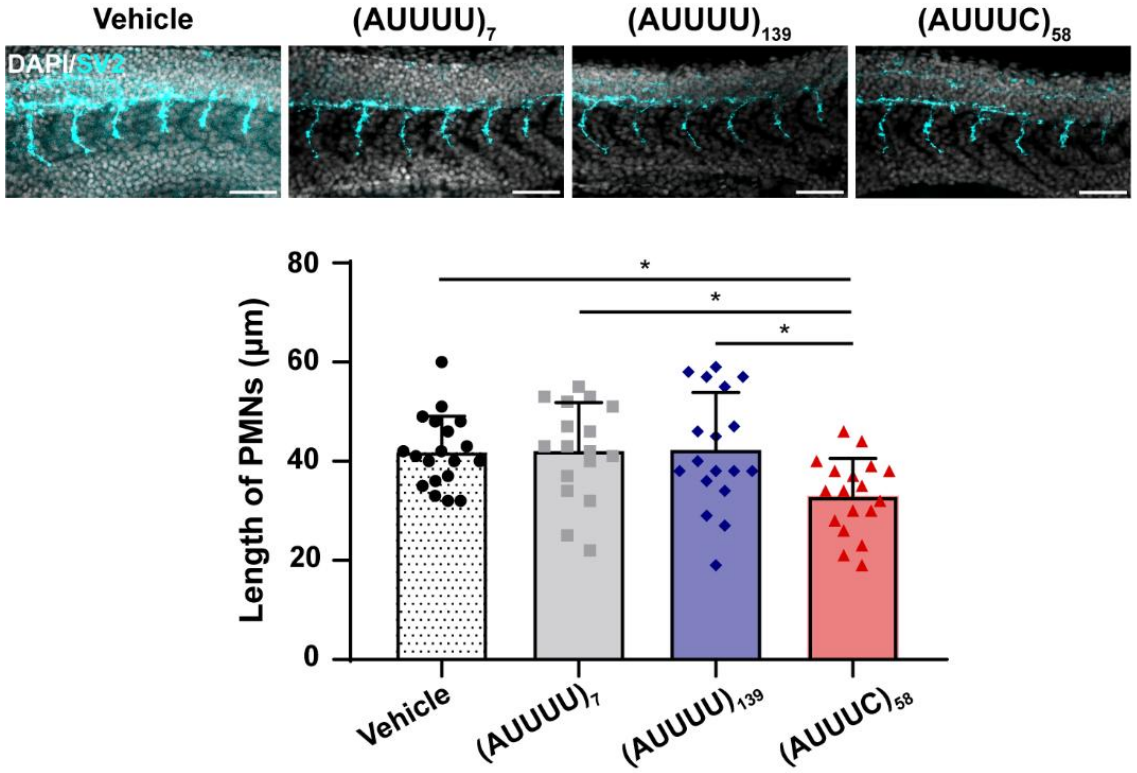

Fig. S7 The large non-pathogenic $(AUUUU)_{139}$ RNA does not induce defective PMN axon outgrowth upon microinjection in zebrafish. Related to **Fig. 3B**. (Top) Representative z-average projections of PMNs axons in the 6-somites region anterior to the cloaca at 24 hpf (scale bar = 50 µm); (bottom) Quantification of PMN axon lengths in zebrafish embryos microinjected with the vehicle, $(AUUUU)_7$, $(AUUUU)_{139}$, or $(AUUUC)_{58}$ RNAs. The sample sizes were as follows: n=19 embryos for vehicle, n=17 embryos for $(AUUUU)_7$, and n=18 embryos for $(AUUUU)_{139}$ and $(AUUUC)_{58}$ RNAs from three experimental replicates. Statistical analysis showed significant differences among conditions (*p<0.05, One-way ANOVA followed by Bonferroni correction).

Disease Models & Mechanisms • Supplementary information

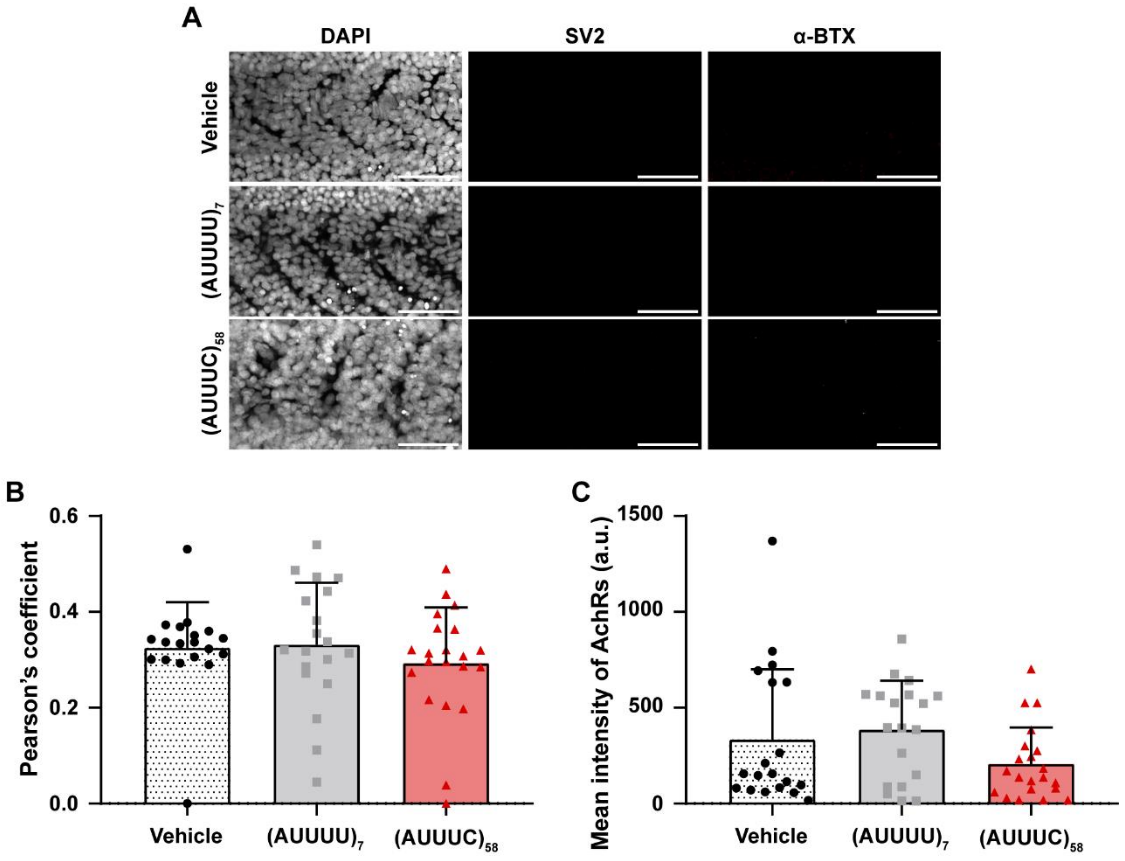

Fig. S8 NMJs in (AUUUC)58-injected zebrafish embryos at 24 hpf. Related to **Fig. 3C.** (A) Representative z-maximum intensity projections showing immunofluorescence without primary antibodies in 24 hpf embryos microinjected with the vehicle, $(AUUUU)_7$ or $(AUUUC)_{58}$ RNAs; scale bar = 50 µm. (B) Co-localization of the presynaptic SV2 and postsynaptic α-BTX markers measured by Pearson's co-localization coefficient, with values ranging from 0 to 1. Data are presented for n=19 embryos in the vehicle and $(AUUUU)_7$ groups, and n=21 embryos in the $(AUUUC)_{58}$ group, sourced from 3 experimental replicates. Statistical analysis was conducted using the Kruskal-Wallis test for the co-localization coefficient, and the results are indicated as not significant. (C) α-BTX mean intensity calculation for NMJs with data collected from n=19 embryos in the vehicle group, n=19 embryos in the $(AUUUU)_7$ group, and n=21 embryos in the $(AUUUC)_{58}$ group, across 3 experimental replicates. Statistical analysis using the Kruskal-Wallis test to assess differences in mean intensity, with results indicated as not significant. Data are represented as mean ± standard deviation.

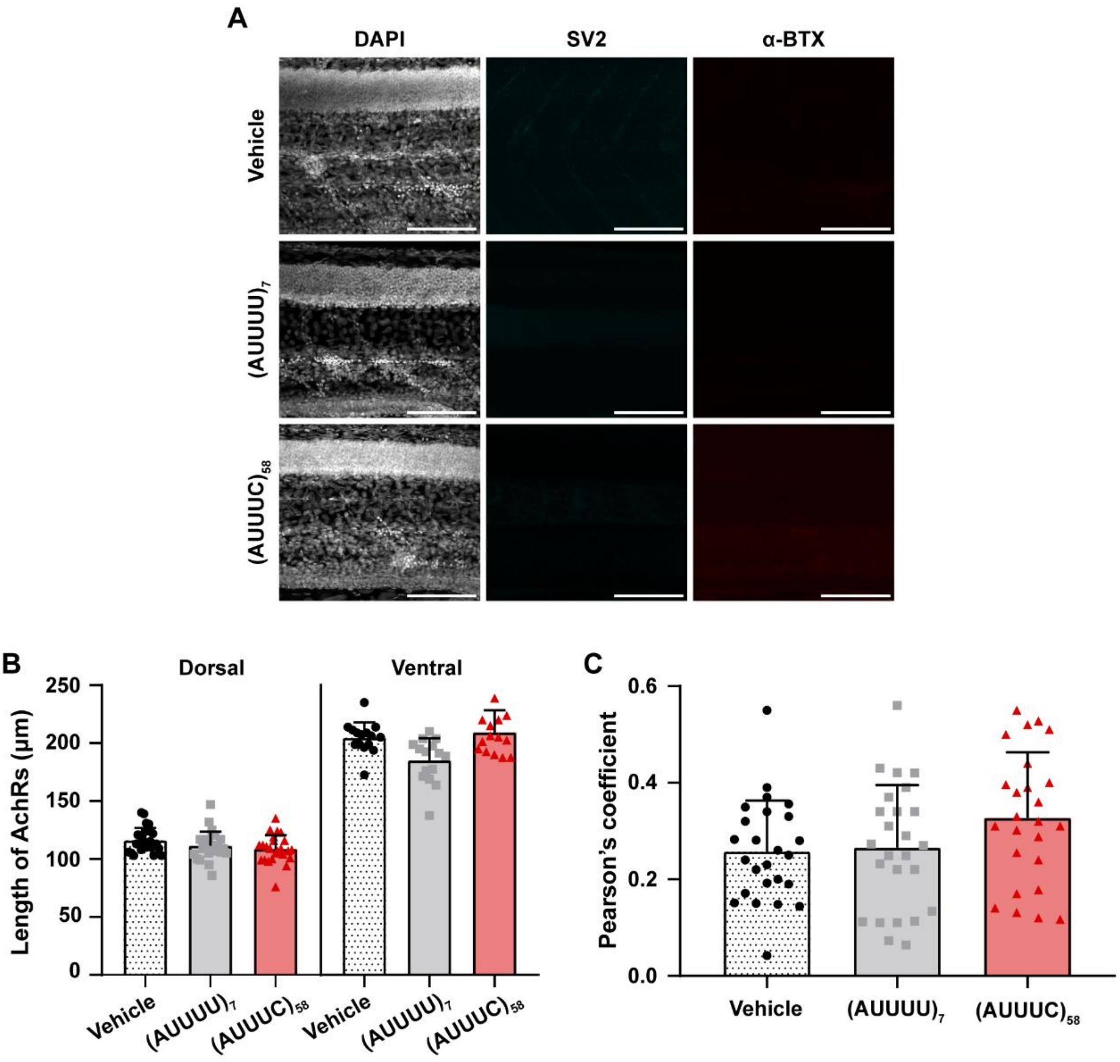

Fig. S9 Analysis of NMJs in $(AUUUC)_{58}$ larvae at 72 hpf. Related to **Fig. 4**. Sample size of 25 larvae per condition from four replicates was used for NMJ analysis at 72 hpf. (A) Z-maximum intensity projections exhibiting immunofluorescence without primary antibodies in larvae previously microinjected with the vehicle, $(AUUUU)_7$ or $(AUUUC)_{58}$ RNAs at 72 hpf; scale bar = 100 µm. (B) Distribution of AchR signal along the dorsal-ventral axis in zebrafish muscle was performed in larvae previously microinjected with vehicle, $(AUUUU)_7$, or $(AUUUC)_{58}$ RNAs. Statistical analysis showed no significant differences among conditions (Kruskal-Wallis test). (C) Co-localization of SV2 and α-BTX was assessed by Pearson's co-localization coefficient. Statistical analysis using the One-way ANOVA test yielded no significant results. Data are presented as mean ± standard deviation.

Disease Models & Mechanisms • Supplementary information

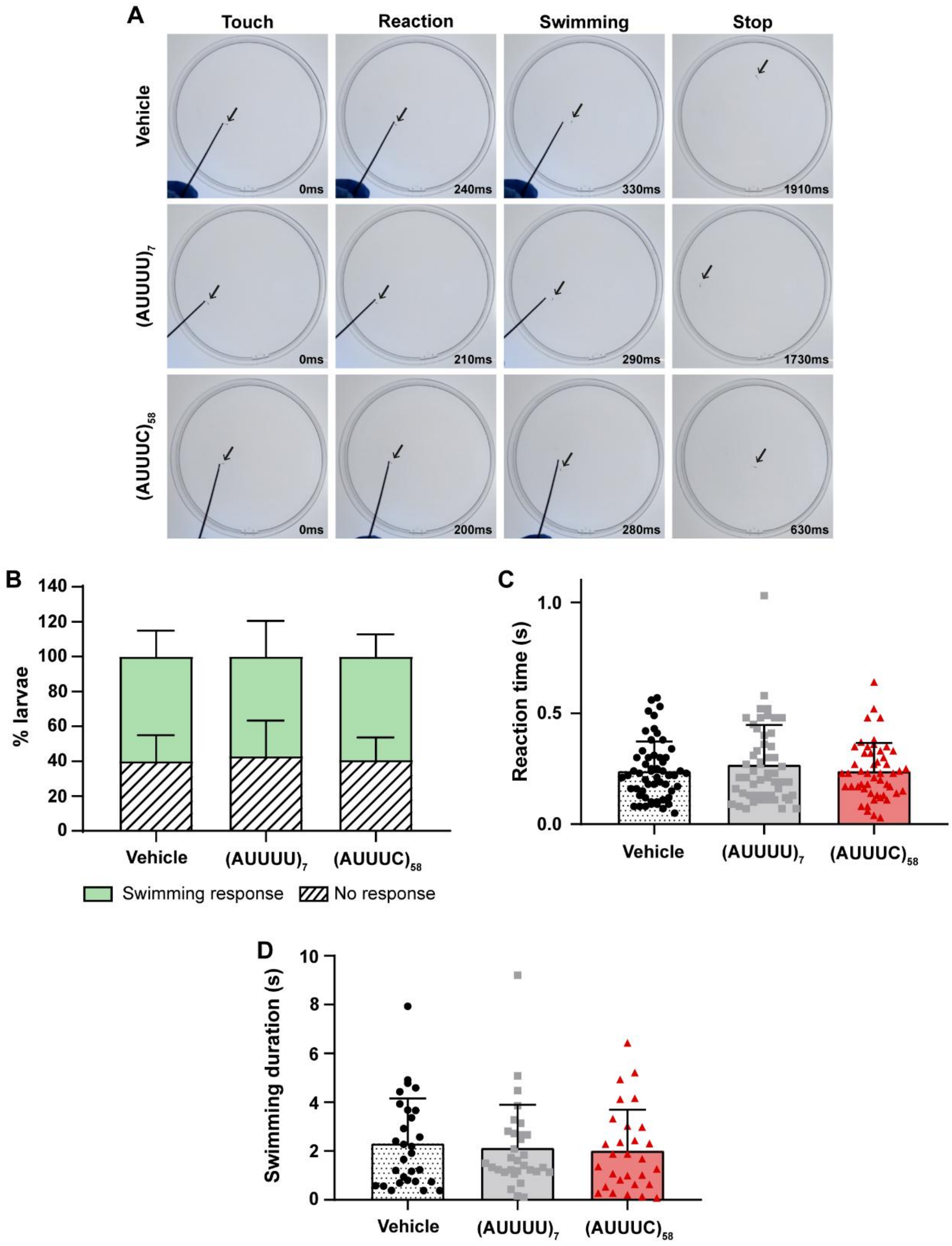

Fig. S10 (AUUUC)$_{58}$ larvae exhibited no motor or sensorial defects. (A) Representative frames showcasing the touch stimuli, the larvae reaction, the swimming away from the filament (10 frames after their reaction) and the cessation of swimming observed during the touch-evoked escape swimming test. This test was performed on larvae microinjected with either the vehicle, (AUUUU)$_7$, or (AUUUC)$_{58}$ at 72 hpf. (B) The data indicates the percentage of larvae that responded or did not respond to the touch stimulus across three different groups: the vehicle

Disease Models & Mechanisms • Supplementary information

group (n=91 larvae), the $(AUUUU)_7$ group (n=88 larvae), and the $(AUUUC)_{58}$ group (n=84 larvae), with a total of six experimental replicates for each condition; χ^2 test for no response or swimming response. (C) The data presents the reaction time of each larva in response to the touch stimulus across three groups: the vehicle group (n=56 larvae), the $(AUUUU)_7$ group (n=53 larvae), and the $(AUUUC)_{58}$ group (n=50 larvae), with a total of six experimental replicates for each condition; Kruskal-Wallis test. (D) The data outlines the swimming duration of each animal following the touch stimulus across three groups: the vehicle group (n=29 larvae), the $(AUUUU)_7$ group (n=31 larvae), and the $(AUUUC)_{58}$ group (n=29 larvae), all derived from six experimental replicates; Kruskal-Wallis test. Data are shown as mean ± standard deviation.

Disease Models & Mechanisms • Supplementary information

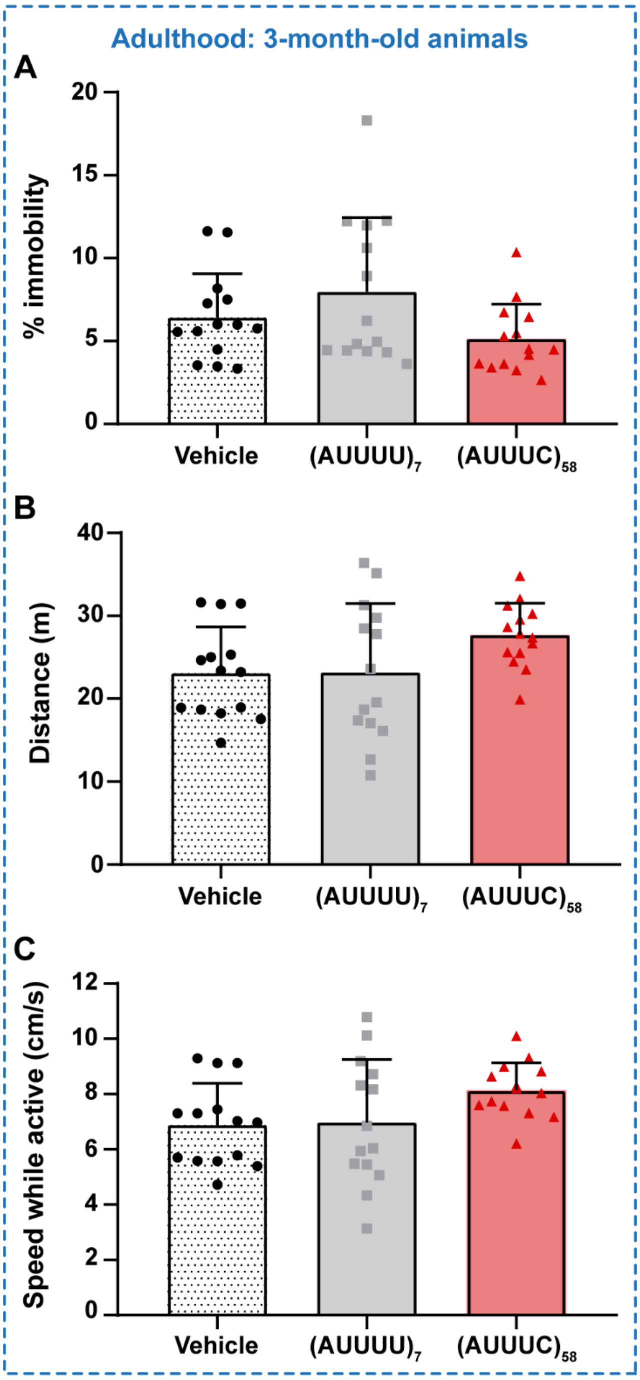

Fig. S11 The 3-month-old (AUUUC)$_{58}$ zebrafish exhibited no motor impairment. Related to **Fig. 6**. The novel diving tank test was conducted on microinjected animals with vehicle, (AUUUU)$_7$, or (AUUUC)$_{58}$ at 3 months of age, with a sample size of 14 animals per condition. (A) Analysis of the percentage of immobility (Kruskal-Wallis test). (B) Analysis of the total swimming distance (Two-way ANOVA with condition and sex as factors for distance). (C) Measurement of the average swimming speed (Two-way ANOVA with condition and sex as factors for speed). Data are shown as mean ± standard deviation.

Disease Models & Mechanisms • Supplementary information

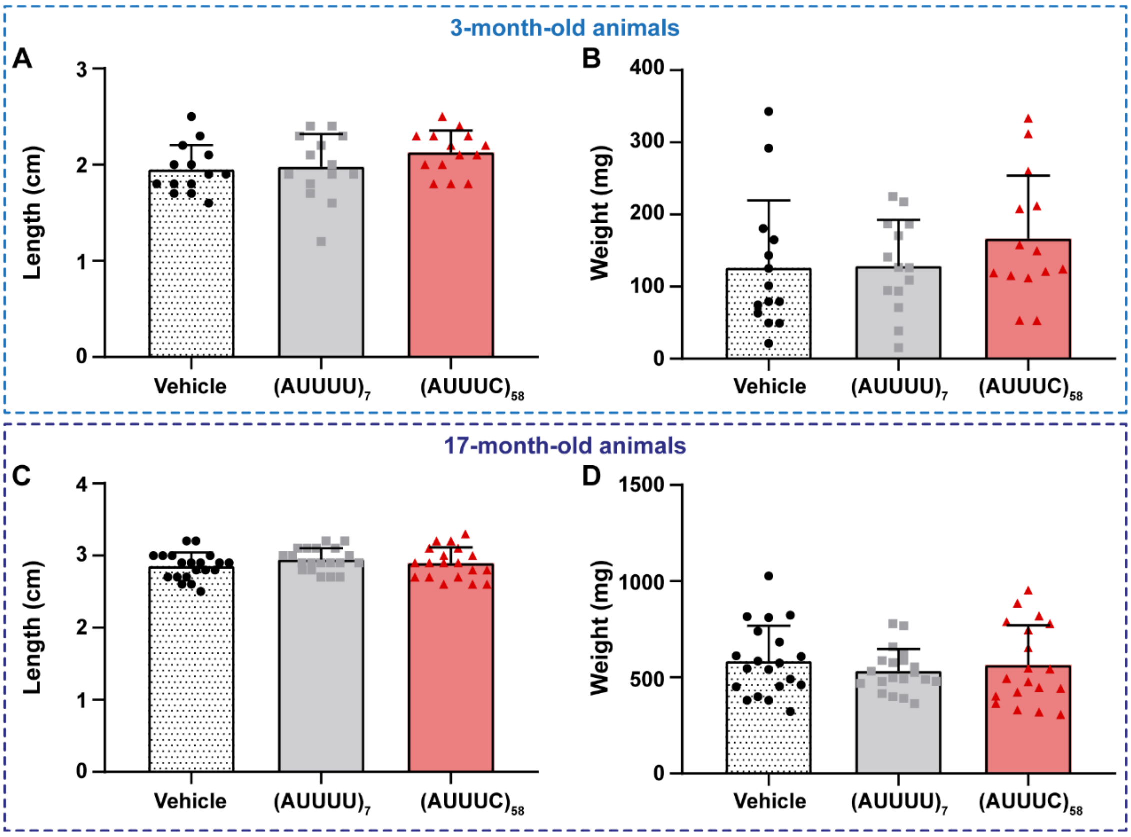

Fig. S12 Comparison of body size and weight in 3- and 17-month-old animals indicates no significant differences. Related to **Fig. 6** and **Fig. S11.** The following sample sizes were used: n=14 animals per condition at 3 months, and n=20 animals for the vehicle and $(AUUUU)_7$ conditions, and n=19 animals for the $(AUUUC)_{58}$ condition at 17 months. The top section presents data for 3-month-old animals, while the bottom section features data for 17-month-old animals. Zebrafish length at 3 months (A) and 17 months (C) was analyzed using a One-way ANOVA, which yielded no significant results. The weight of zebrafish assessed at 3 months (B) and 17 months (D) was evaluated using the Kruskal-Wallis test and One-way ANOVA test, respectively, showing no significant differences. Data are represented as mean ± standard deviation.

Disease Models & Mechanisms • Supplementary information

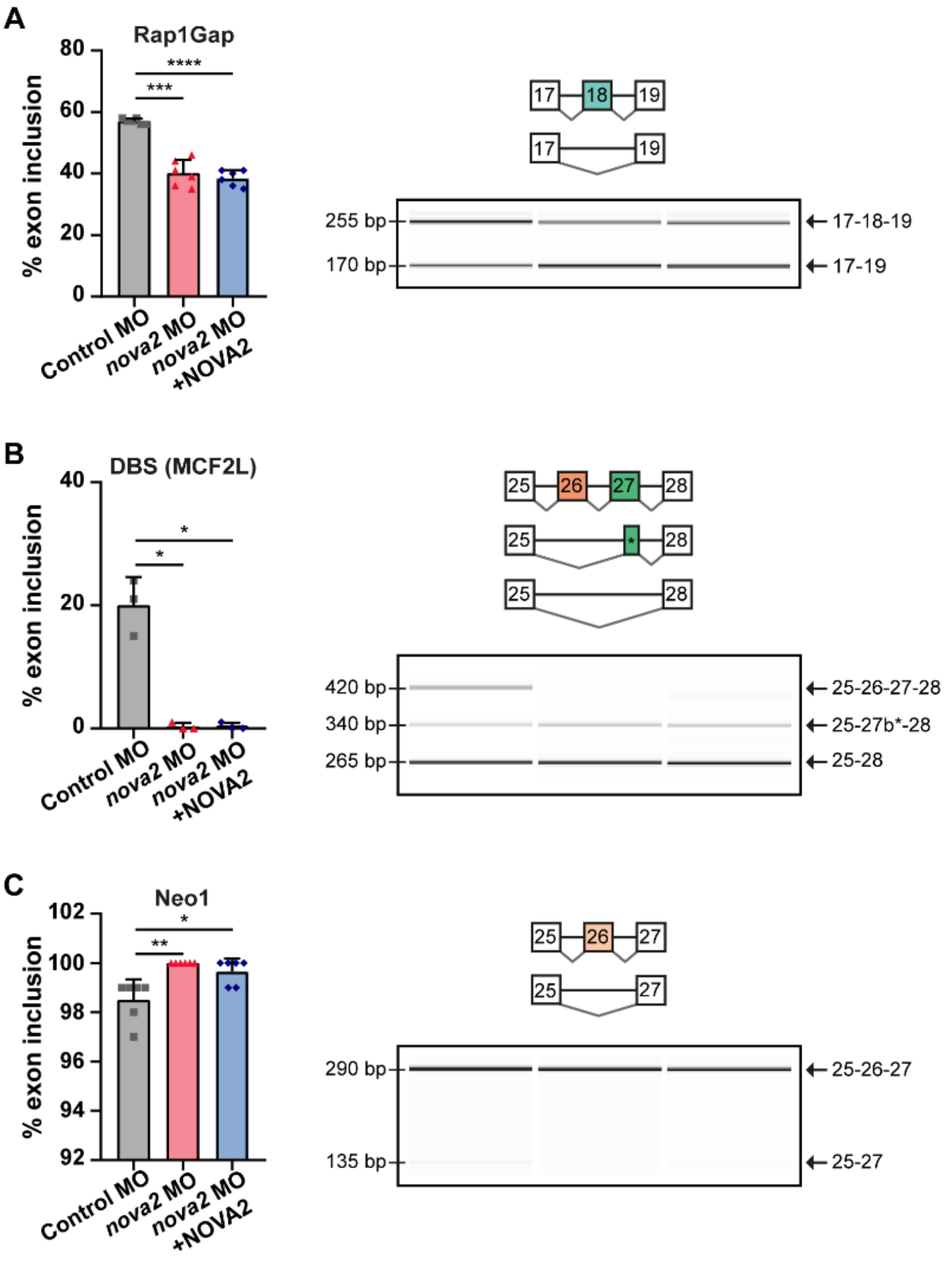

Fig. S13 Validation of *nova2* morpholino-mediated knockdown in zebrafish. Related to **Fig. 8A**. The percentage of exon inclusion in Nova2 pre-mRNA targets at 24 hpf after microinjection of control, *nova2,* or a combination of *nova2* morpholino and human *NOVA2* mRNA. Transcripts alternatively spliced by Nova2 were amplified by RT-PCR, analyzed by electrophoresis on 2100

Disease Models & Mechanisms • Supplementary information

Bioanalyzer (Agilent), and confirmed by sequencing. Statistical analysis revealed significant differences in the inclusion of the exons highlighted between control and *nova2* morpholino-injected embryos for (A) *Rap1Gap* (***$p<0.001$ and ****$p<0.0001$, One-way ANOVA followed by Welch correction and Dunnett's T3 post-hoc test, 6 biological replicates); (B) *DBS* (Asterisk corresponds to a alternatively spliced variant containing the exon 27b, previously identified by Giampietro and colleagues; *$p<0.05$, One-way ANOVA followed by Welch correction and Dunnett's T3 post-hoc test, 3 biological replicates); and, (C) *Neo1* (*$p<0.05$ and **$p<0.01$; Kruskal-Wallis test followed by Dunn's post-hoc test, 6 biological replicates). No significant differences were observed between embryos microinjected with *nova2* morpholino and *nova2* morpholino + human *NOVA2* mRNA. Data are shown as mean ± standard deviation. Abbreviations: MO – morpholino.

Table S1. Maximum Z-scores for RBPs predicted to bind SCA37 $(AUUUC)_{58}$ RNA, as determined by the RBPmap webserver. Related to **Fig. 7A**.

RBP	Non-pathogenic RNA	SCA37 RNA
A1CF	3.267	3.267
ANKHD1	2.551	2.551
BOLL	3.829	3.829
BRUNOL4	3.865	3.865
BRUNOL5	3.85	3.85
BRUNOL6	3.16	3.16
CELF1	3.629	3.629
CNOT4	2.436	2.436
CPEB1	3.29	3.29
CPEB2	3.929	3.929
CPEB4	3.696	3.696
DAZ3	3.721	3.721
DAZAP1	2.903	2.903
EIF4G2	2.727	2.727
ELAVL4	3.67	3.67
EWSR1	1.991	1.991
FUBP1	3.44	3.44
FUBP3	3.562	3.562
FXR1	1.7	1.7
G3BP2	2.053	2.053
HNRNPA0	3.18	3.18
HNRNPA1	3.311	3.311
HNRNPA1L2	1.887	1.887
HNRNPA2B1	2.745	2.745
HNRNPC	3.634	3.634
HNRNPCL1	3.658	3.658
HNRNPD	2.988	2.988
HNRNPDL	3.152	3.152
HNRNPF	2.361	2.361
HNRNPH1	1.93	1.93
HNRNPL	3.013	3.013
HNRNPM	2.159	2.159
HNRNPU	3.031	3.031
HNRPLL	2.861	2.861
HuR	3.857	3.857
IGF2BP1	1.761	1.761
IGF2BP2	3.466	3.466
IGF2BP3	3.373	3.373
KHDRBS1	2.928	2.928
KHDRBS2	2.788	2.788

Disease Models & Mechanisms • Supplementary information

Table S1 (continued)

RBP	Non-pathogenic RNA	SCA37 RNA
KHDRBS3	2.972	2.972
KHSRP	3.282	3.282
LIN28A	2.5	2.5
MATR3	2.703	2.703
MBNL1	2.903	2.903
MSI1	2.476	2.476
NOVA1	2.65	4
NUPL2	2.636	2.636
PABPC1	3.235	3.235
PABPC3	3.216	3.216
PABPC4	3.327	3.327
PABPC5	3.068	3.068
PABPN1	2.528	2.528
PABPN1L	3.094	3.094
PCBP1	2.044	2.044
PCBP2	3.037	3.037
PCBP3	3.635	3.635
PCBP4	2.806	2.806
PRR3	2.274	2.274
PTB3	3.296	3.296
PTBP3	3.337	3.337
PUF60	3.403	3.403
PUM1	3.211	3.211
PUM2	2.966	2.966
QKI	2.207	2.207
RALY	4.17	4.17
RBFOX1	3.039	3.039
RBM15B	4.319	4.319
RBM23	2.9	2.9
RBM24	4.323	4.323
RBM28	2.514	2.514
RBM38	3.974	3.974
RBM41	3.42	3.42
RBM42	2.811	2.811
RBM45	3.089	3.089
RBM47	2.78	2.78
RBM6	3.139	3.139
RBMS1	3.836	3.836
RBMS2	3.19	3.19
RBMS3	3.656	3.656
RC3H1	3.535	3.535
SAMD4A	1.716	1.716

Table S1 (continued)

RBP	Non-pathogenic RNA	SCA37 RNA
SART3	3.235	3.235
SF1	2.72	2.72
SFPQ	2.731	2.731
SNRNP70	1.767	1.767
SNRPA	2.237	2.237
SRSF1	2.839	2.839
SRSF10	2.787	2.787
SRSF11	1.868	1.868
SRSF2	3.375	3.375
SRSF4	1.842	1.842
SRSF5	2.763	2.763
SRSF8	3.463	3.463
SRSF9	2.743	2.743
TARDBP	2.99	2.99
TIA1	4.127	4.127
TRA2A	2	2
TRNAU1AP	3.988	3.988
TUT1	1.836	1.836
U2AF2	3.571	3.571
UNK	2.672	2.672
YBX1	3.571	3.571
YBX2	3.608	3.608
ZC3H14	4.062	4.062
ZCRB1	2.531	2.531
ZFP36	4	4
ZNF326	2.975	2.975
ZNF638	3.108	3.108

RBP – RNA-binding protein; NOVA1 highlighted in red; $p<0.05$

Table S2. List of primers used in PCR experiments.

Primer	Sequence
**Amplification of RNA repeats**	
STR24 GXL F1	AGGCAGAGGCAGGTGGACCACTTTAG
STR24 GXL R	CTCCCAAGTCAGCCTCCCAGGTAAC
**Amplification of human NOVA2**	
His-tag_Fw•	CATCATCATCATCATCATTGAGGCCTGTGGTGTGTGCTC
His-tag_Rv•	ATGATGATGATGATGATGTCCCACTTTCTGGGGGTTTGAGG
**Detection of Nova2 splicing targets**	
Dock6_Fw*	CCACATCAAGACCCAGTGC
Dock6_Rv*	CCACTGCAGAGCCAACTCTT
DBS_Fw*	CCGATCAGATCTCCCCATTA
DBS_Rv*	TTTCTTCCCTCCCTCATCCT
Neo1_Fw+	TGATGCCCTTTGATGCACAAC
Neo1_Rv+	GAGCTGTGAAAGCTAATGTCCGTC
Rap1gap_Fw*	TGGGCCTCAATATCAGGAAG
Rap1gap_Rv*	CTACGCCGAGAACTGAAAGG

Primers from Figueiredo et al. 2025 (•); Giampietro et al. 2015 (*) and from Jelen et al. 2007 (+).

Table S3 Sequences of morpholinos microinjected in zebrafish embryos.

Oligonucleotide	Sequence
**Morpholinos**	
Standard control morpholino	CCTCTTACCTCAGTTACAATTTATA
nova2 morpholino	TGCACTGCCCCACCGGCCATCATTT

Morpholinos from Giampietro et al. 2015.

Disease Models & Mechanisms • Supplementary information

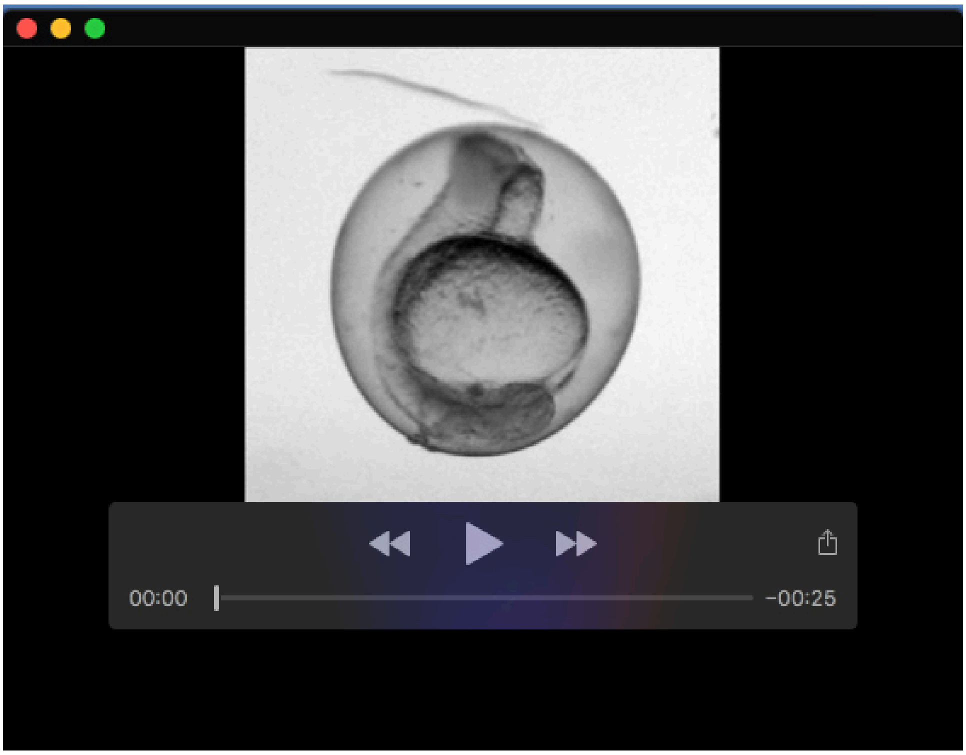

Movie 1. Representative video file capturing spontaneous tail coil movements in a zebrafish embryo microinjected with vehicle control.

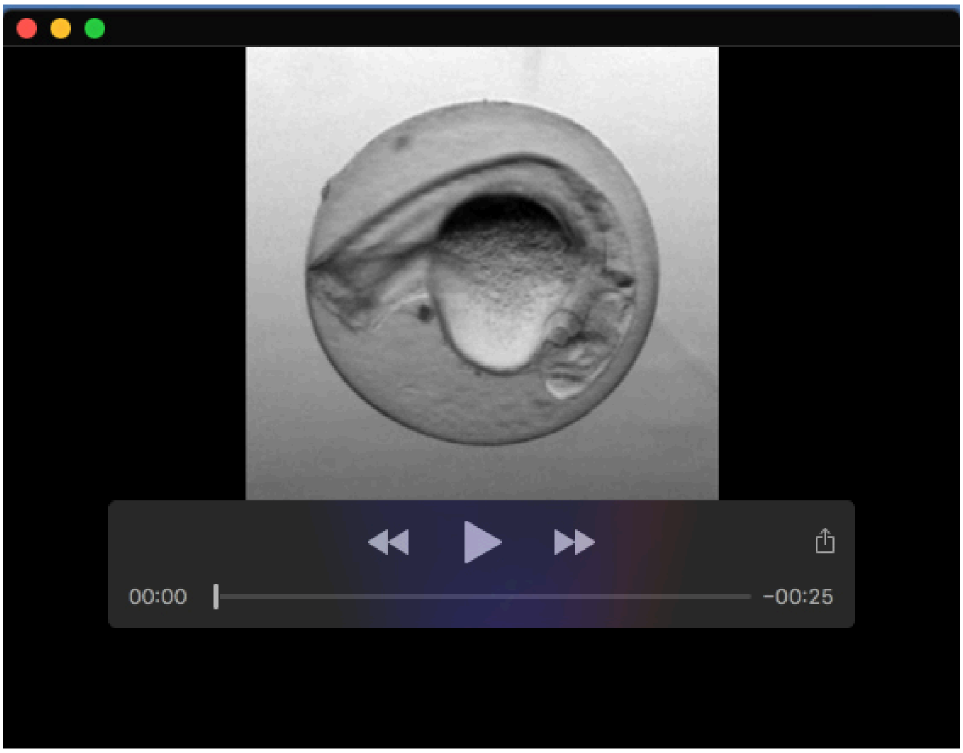

Movie 2. Representative video file capturing spontaneous tail coil movements in a zebrafish embryo microinjected with the non-pathogenic $(AUUUU)_7$ RNA control.

Disease Models & Mechanisms • Supplementary information

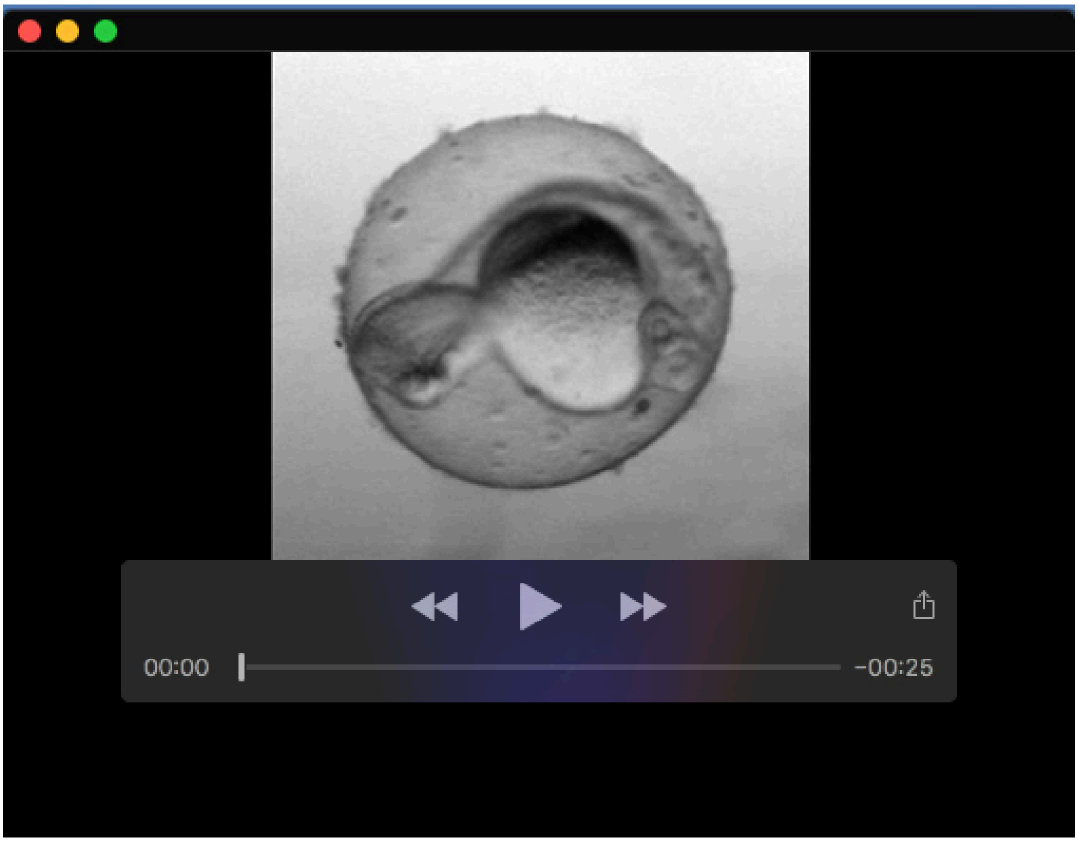

Movie 3. Representative video file capturing spontaneous tail coil movements in a zebrafish embryo microinjected with the pathogenic $(AUUUC)_{58}$ RNA.
